# Evaluation of the Integrated Intervention for Dual Problems and Early Action Among Latino Immigrants With Co-occurring Mental Health and Substance Misuse Symptoms: A Randomized Clinical Trial

**DOI:** 10.1001/jamanetworkopen.2018.6927

**Published:** 2019-01-11

**Authors:** Margarita Alegría, Irene Falgas-Bague, Francisco Collazos, Rodrigo Carmona Camacho, Sheri Lapatin Markle, Ye Wang, Enrique Baca-García, Benjamin Lê Cook, Ligia M. Chavez, Lisa Fortuna, Lizbeth Herrera, Adil Qureshi, Zorangeli Ramos, Claudia González, Paloma Aroca, Lucía Albarracín García, Lucía Cellerino, Ana Villar, Naomi Ali, Kim T. Mueser, Patrick E. Shrout

**Affiliations:** 1 Department of Medicine and Psychiatry, Harvard Medical School, Boston, Massachusetts; 2 Disparities Research Unit, Department of Medicine, Massachusetts General Hospital, Boston; 3 Department of Psychiatry and Forensic Medicine, Autonomous University of Barcelona, Barcelona, Spain; 4 Department of Psychiatry, Hospital Universitari Vall d'Hebron, Barcelona, Spain; 5 Psychiatry Department, Autonoma University of Madrid, Madrid, Spain; 6 Department of Psychiatry, University Hospital Rey Juan Carlos, Mostoles, Madrid, Spain; 7 Department of Psychiatry, General Hospital of Villalba, Villalba, Madrid, Spain; 8 Department of Psychiatry, University Hospital Infanta Elena, Valdemoro, Madrid, Spain; 9 Centro de Investigación en Salud Mental (CIBERSAM), Carlos III Institute of Health, Madrid, Spain; 10 Department of Psychiatry, Universidad Católica del Maule, Talca, Chile; 11 Department of Psychiatry, Instituto de Investigación Sanitaria, Fundación Jiménez Díaz, Madrid, Spain; 12 Department of Psychiatry, Harvard Medical School, Boston, Massachusetts; 13 Behavioral Sciences Research Institute, Medical Sciences Campus, University of Puerto Rico, San Juan, Puerto Rico; 14 Boston Medical Center, Department of Psychiatry, Boston University School of Medicine, Boston, Massachusetts; 15 Biomedical Network Research Centre on Mental Health, CIBERSAM, University of Barcelona, Barcelona, Spain; 16 Center for Psychiatric Rehabilitation, Boston University, Boston, Massachusetts; 17 Department of Psychology, New York University, New York City

## Abstract

**Importance:**

Immigrants are at an increased risk for co-occurring mental health and substance misuse symptoms; however, effective treatments are lacking.

**Objective:**

To evaluate the effectiveness of the Integrated Intervention for Dual Problems and Early Action (IIDEA) program compared with enhanced usual care.

**Design, Setting, and Participants:**

This effectiveness randomized clinical trial was conducted from September 2, 2014, to February 2, 2017, in 17 clinics or emergency departments and 24 community sites in Boston, Massachusetts, as well as in Madrid and Barcelona, Spain. Equal randomization (1:1) in 2-person blocks was used, assigning participants to either the IIDEA treatment group (n = 172) or the enhanced usual care control group (n = 169). Intent-to-treat analyses assessed effectiveness, and post hoc analyses examined whether results varied by symptom severity or treatment dose. Eligible participants were between 18 and 70 years of age, self-identified as Latino, screened positive for co-occurring symptoms, and were not receiving specialty behavioral health services.

**Interventions:**

Participants were randomized to a 10-session IIDEA treatment or to enhanced usual care.

**Main Outcomes and Measures:**

Primary outcomes were changes in alcohol and drug misuse and results of a urine test for drug metabolites but not for alcohol misuse. Secondary outcomes were symptoms of depression, generalized anxiety, posttraumatic stress disorder, and overall mental health.

**Results:**

In total, 341 participants were randomized to either the IIDEA treatment group (n = 172; 94 [54.7%] female, mean [SD] age, 33.5 [11.6] years) or the enhanced usual care control group (n = 169; 80 [47.3%] female, mean [SD] age, 34.3 [11.8] years). No statistically significant effects of IIDEA were found for primary drug and alcohol outcomes (ASI Lite–drug score: β = −0.02 [SE, 0.69; *P* = .88; Cohen *d*, 0.00; 95% CI, −0.17 to 0.17]; ASI Lite–alcohol score: β = −0.01 [SE, 1.19; *P* = .66; Cohen *d,* 0.00; 95% CI, −0.12 to 0.12]; urine drug test result: β = −0.36 [SE, 0.43; *P* = .50; OR, 0.70; 95% CI, 0.30-1.61]), but statistically significant effects were observed for secondary mental health outcomes. The IIDEA treatment was effective in reducing depressive symptoms per the Patient Health Questionnaire-9 score (β = −1.14; SE, 0.47; *P* = .02; Cohen *d*, 0.20 [95% CI, 0.04-0.36]), posttraumatic stress disorder symptoms per the Posttraumatic Stress Disorder Checklist-5 score (β = −3.23; SE, 1.59; *P* = .04; Cohen *d*, 0.25 [95% CI, 0.01-0.37]), and overall mental health symptoms per the Hopkins Symptom Checklist-20 (β = −0.20; SE, 0.07; *P* = .01; Cohen *d*, 0.25 [95% CI, 0.08-0.42]) and composite mental health (β = −3.70; SE, 1.75; *P* = .04; Cohen *d*, 0.19 [95% CI, 0.01-0.36]) scores at the 6-month follow-up. Exploratory analyses suggested that 6-month treatment effects occurred for patients whose drug misuse was moderate to severe at the baseline assessment. Among patients with moderate to severe substance misuse, IIDEA substantially reduced substance use per the urine test results (odds ratio, 0.25 [95% CI, 0.09-0.67]; *P* = .01). Treatment dose showed small to large effect sizes by outcome.

**Conclusions and Relevance:**

The IIDEA treatment did not change drug misuse but did improve secondary mental health and substance misuse outcomes for a heterogeneous population with moderate to severe symptoms; this finding provides a path for treating Latino immigrants with co-occurring mental health and substance misuse symptoms.

**Trial Registration:**

ClinicalTrials.gov Identifier: NCT02038855

## Key Points

**Question:**

Would a tailored behavioral health intervention reduce substance misuse and mental health symptoms, compared with enhanced usual care, in Latino immigrants with co-occurring mental health and substance misuse symptoms?

**Findings:**

In this randomized clinical trial from 3 sites of 341 immigrants with co-occurring mental health and substance misuse symptoms, the primary outcome of substance misuse did not change in the intent-to-treat analysis. Patients who received the treatment statistically significantly experienced decreased mental health symptoms, compared with controls under enhanced usual care, and only participants with moderate to severe symptoms who received the intervention statistically significantly reduced their substance misuse.

**Meaning:**

The intervention did not change drug misuse in a heterogeneous sample but did improve secondary mental health outcomes, a finding that might provide a path for treating Latino immigrants with co-occurring mental health symptoms whose symptoms are in the moderate-to-severe range.

## Teaser

This randomized clinical trial evaluates the effectiveness of an integrated, multiyear cognitive behavior therapy, the Integrated Intervention for Dual Problems and Early Action (IIDEA), compared with enhanced usual care among Latino immigrants with dual mental health and substance misuse symptoms.

## Introduction

Immigrants in the United States, for example, are at risk for co-occurring mental health and substance misuse symptoms.^1^ However, we know little about whether treatments that are effective for native-born populations are also helpful for foreign-born populations with co-occurring symptoms. Patients with co-occurring symptoms have more severe impairment; worse treatment outcomes; higher morbidity and mortality; increased treatment costs; and greater risk for homelessness, incarceration, and suicide than patients with either a mental health or substance misuse disorder alone.^2,3,4^ Because less than half of individuals with co-occurring symptoms access treatment,^5^ engagement of immigrants with co-occurring symptoms is a serious obstacle, given their fears of deportation, high rates of uninsurance, linguistic barriers, and discrimination.^6,7^ Identifying effective treatments for Spanish-speaking immigrants is particularly important, as Latino populations (eg, people from predominantly Spanish-speaking countries in North, Central, or South America and the Caribbean islands) are the largest and fastest-growing immigrant population in the United States^8^ and Spain,^9^ and they confront enormous barriers to accessing behavioral health treatments.^10^ Structural and institutional barriers^6^ mean that Latino individuals may be less likely to access evidence-based practices^11^ or have poorer clinical outcomes than non-Latino individuals.^12^

The literature suggests that Latino immigrants with co-occurring symptoms could be well served by an intervention that is appropriate for a heterogeneous population, requires limited screening and resources, and is relevant to those with diverse symptom severity.^13,14^ Thus, we developed the Integrated Intervention for Dual Problems and Early Action (IIDEA) program, a cognitive-restructuring, mindfulness-based therapy that includes substance-craving reduction and coping strategies, designed to provide culturally tailored and evidence-based care for Latino populations with co-occurring symptoms. We assessed IIDEA’s effectiveness and compared it with the effectiveness of enhanced usual care in reducing substance misuse and improving mental health symptoms among Latino individuals with co-occurring mental health symptoms (depression, anxiety, and trauma symptoms) and substance misuse (use of drugs, alcohol, and prescription medication for a purpose not consistent with medical or legal guidelines).^15^ We describe the results of this multisite randomized clinical trial conducted in Spain (Madrid and Barcelona) and the United States (Boston, Massachusetts). We hypothesized that patients who received the IIDEA program would show symptom reduction and a greater likelihood of substance use abstinence compared with those receiving enhanced usual care.

## Methods

### Study Design

This effectiveness trial^16^ used equal randomization (1:1) to the IIDEA treatment or to enhanced usual care and was conducted from September 2, 2014, to February 2, 2017, in 17 clinics or emergency departments and 24 community sites in Boston, Madrid, and Barcelona. The trial protocol is available in Supplement 1. This study followed the Consolidated Standards of Reporting Trials (CONSORT) guidelines, including the checklist and diagram to track participants during the enrollment and trial procedures (Figure). Human participation approval was obtained from the institutional review boards of all 3 participating institutions (Massachusetts General Hospital, Vall d’Hebron University Hospital, and Fundación Jimenez Diaz). All participants provided written informed consent.

**Figure.**
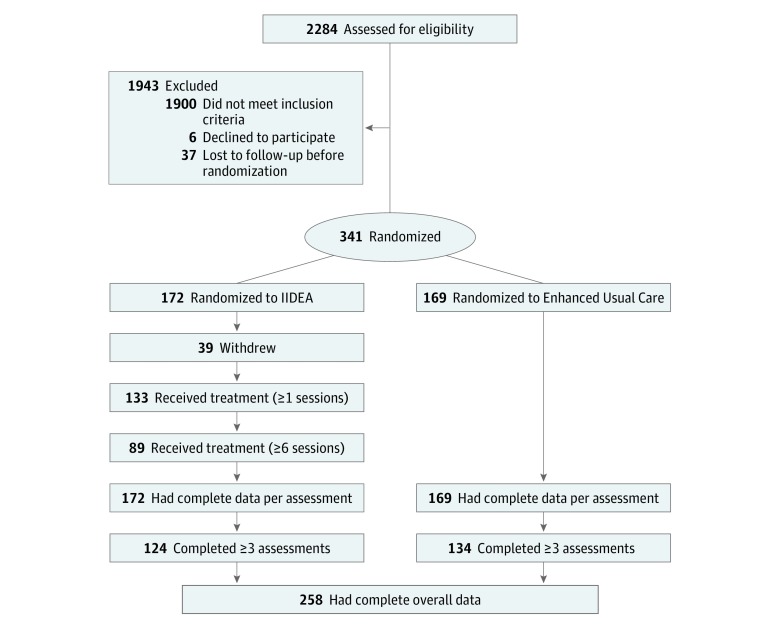
CONSORT Flow Diagram IIDEA indicates Integrated Intervention for Dual Problems and Early Action.

Treatment consisted of 10 to 12 sessions, lasting 45 to 75 minutes each, of the IIDEA cognitive behavioral intervention, which was adapted for diverse Latino patients; details of the cultural adaptation of IIDEA are included in eAppendix 2 in Supplement 2. Trained clinicians at the sites delivered the treatment for 3 to 6 months in person or by telephone, and the sessions were audio-recorded for quality control. A care manager at the sites contacted the enhanced usual care group to assess their symptoms, ensure safety, and assist with referrals. Patients in the enhanced usual care group met with their primary care physician as usual, if they were in care. Research assistants, who were blind to study randomization, assessed the outcomes (including results of a urine drug screen) at 2, 4, 6, and 12 months after baseline between November 3, 2014, and February 28, 2017. Participants received $30 (in the United States) or €25 (in Spain) for each assessment, and the incentives increased to $50 (or €30) for the 6- and 12-month assessments. An independent advisory board was consulted annually during the study period. For quality control purposes, data collection supervisors (L.H., L.C., P.A., and A.V.)****listened to the first 3 interviews for each research assistant and a random 15% of the sample. Detailed information on the study design is in eFigure 1 and eAppendix 1 in Supplement 2.

### Participants and Recruitment Sites

Bilingual (English and Spanish) study staff enrolled participants through direct contact in primary care clinic waiting rooms, emergency departments, and Latino-serving community-based organizations or through referrals by health care professionals or other participants. We met with clinic- or community-based organization leaders and staff to present our study and tailor recruitment to each site.

Eligible participants were 18 to 70 years of age, self-identified as Latino, screened positive for co-occurring symptoms, and were not receiving or about to receive specialty behavioral health services (ie, therapy sessions with a psychiatrist, psychologist, or social worker) in the previous 3 months or the upcoming month. Positive co-occurring screening symptoms included an affirmative response to 2 questions about mental health and 2 about substance misuse on the AC-OK (AC [Cherry and Dillon], OK [Oklahoma])^17^ screener, a 15-item questionnaire validated in Spanish. This cutoff point provided 0.95 sensitivity, 0.62 specificity, and 0.90 area under the curve for mental health as well as 0.68 sensitivity, 0.90 specificity, and 0.83 area under the curve for substance misuse, when compared with validated screener measures.^18^ The same cutoff point provided 0.67 sensitivity and 0.74 specificity for screening co-occurring symptoms.

Potential participants were excluded if they lacked the capacity to consent (assessed by a validated screener)^19^ or reported imminent suicidal ideation (assessed with the Paykel Suicide Scale).^20^ Those excluded for suicidality were clinically assessed and referred to urgent care, with the opportunity to rescreen after a 30-day period. When research assistants detected cognitive impairment associated with intoxication or severe substance use during the screening and/or assessments, they included a note in the data and reported the details to study supervisors. The research team discussed possible referral for detoxification services at study supervision meetings and followed each case to determine if the individual could potentially take part in the trial once stabilized.

### Randomization and Masking

A study investigator (B.L.C.) generated a stratified block-randomization scheme^21^ that assigned eligible participants to the treatment or enhanced usual care groups in a 1:1 ratio for each 2-person block. Stratified by recruitment site and then clinician, each patient had a 50% chance of being assigned to the intervention condition. The project coordinator at each site (who was not involved in data collection) randomized patients after obtaining informed consent and baseline assessment. Research assistants, blinded to the study condition, administered the follow-up assessments. Care managers and clinicians were not blinded.

### Enhanced Usual Care and IIDEA Groups 

Participants in the enhanced usual care group continued usual care with their primary care physician, if available. A care manager contacted enhanced usual care participants once every 3 weeks during the treatment period (5 calls within a 6-month period) to administer the same assessment used in the treatment sessions. This monitoring ensured that participants were not deteriorating, or if they were, the care manager assisted them with referrals to mental health or social services.

The IIDEA intervention is a transdiagnostic manualized therapy that addresses elevated mental health symptoms and symptoms of drug, alcohol, and benzodiazepine misuse; in this trial, this intervention was tailored specifically for Latino individuals. The intervention integrates cognitive behavioral therapy, motivational interviewing, mindfulness practice, and cognitive restructuring. The substance misuse component includes strategies for reducing cravings, preventing relapse, strengthening coping skills, and preventing HIV or risk for sexually transmitted infections. The IIDEA treatment concludes with the creation of a self-care plan, practice of learned skills, and additional booster sessions if needed. In this trial, IIDEA treatment was conducted in the participant’s preferred language (English or Spanish) and was offered at home if there were childcare or illness constraints. Detailed information can be found in eAppendix 2 in Supplement 2.

### Therapists and Treatment Fidelity

The intervention was delivered weekly or biweekly by clinicians with a master’s degree or higher (psychiatry residents, psychologists, social workers, and counselors). Clinician training consisted of 50 hours of didactic instruction and role play. Supervisors rated the first 2 sessions and a random 15% of the total sample of sessions on a standardized fidelity scale (eAppendix 2 in Supplement 2). Clinician fidelity was 84.8%. Weekly telephone supervisor meetings facilitated case review and treatment fidelity across the sites, with an interrater agreement of 94.6% among 9 supervisors (eAppendix 2 in Supplement 2).

### Outcome Measures

Primary outcome measures were changes in the drug (α = .84; score range, 0-1; cutoff score, >0.1) and alcohol (α = .70; score range, 0-1; cutoff score, >0.1) components of the Addiction Severity Index (ASI) Lite,^22^ which evaluates lifetime and past 30-day behaviors, as well as changes in urine drug test findings (DrugCheck Nx; TransMed),^23^ which checks for a binary outcome of drug metabolites and use of any 6 drug types (amphetamine, benzodiazepine, cocaine, methamphetamine, heroin, and marijuana) but not for alcohol misuse. Secondary outcome measures were the Patient Health Questionnaire (PHQ-9) (α = .85; score range, 0-27; cutoff score, >10) for depressive symptoms^24^; the Generalized Anxiety Disorder Scale (GAD-7) (α = .86; score range, 0-21; cutoff score, >10)^25^ for generalized anxiety symptoms; the Posttraumatic Stress Disorder (PTSD) Checklist (PCL-5) (α = .94; score range, 0-80; cutoff score, >33)^26^ for PTSD symptoms; and the Hopkins Symptom Checklist (HSCL-20) (α = .94; score range, 0-4; cutoff score, >1.5)^27^ for overall mental health symptoms. All of these measures have been validated in both English and Spanish^28,29^ (eTable 1 in Supplement 2 provides a detailed description of each instrument).

We created a composite measure for substance use symptoms, using the Alcohol Use Disorders Identification Test (α = .78; score range, 0-12),^30^ Drug Abuse Screening Test (α = .87; score range, 0-10),^31^ and selected items with high internal consistency from the Benzodiazepine Dependence Questionnaire (α = .90; score range, 0-27)^32^ as well as a composite construct for mental health, using PHQ-9, GAD-7, and PCL-5. Each component measure was standardized to a scale of 0 to 100, and the composite was formed on the basis of their mean. The cutoff scores for mental health were 35 points or higher and for substance misuse 20 points or higher. We updated 2 measures from the originally registered protocol (eTable 1 in Supplement 2). We also collected demographic data and other social and cultural characteristics of the participants for secondary analysis (eTable 1 in Supplement 2).

### Statistical Analysis

The patient sample size (n = 360) was chosen to achieve adequate power to detect meaningful effect sizes on the primary outcomes.^33^ The actual sample (n = 341) approximates the target sample size with attrition. We used effect sizes from similar studies with Latino populations and a pilot study^34^ to estimate the sample needed to have 80% power to detect substantial treatment effects. Our primary analyses used intent-to-treat principles and focused on treatment effects at intervention completion (at most, 6 months after baseline). Effect sizes were calculated in the metric of odds ratio (OR) for binary outcomes and in the metric of Cohen *d* for continuous outcomes. The ORs were calculated as an exponent of the regression coefficient from the estimated logit model. Approximate 95% CIs are reported here, assuming the regression coefficients are normally distributed approximately.

The 4 postrandomization assessments were included in multilevel mixed models. To account for missing data or incomplete assessments, we used multiple imputations via chained equations (eAppendix 3 and eTable 5 in Supplement 2).^21^ The mixed model included a term to account for linear change in outcomes, a different slope between 6-month and 12-month assessments, and a random effect for intercept (eAppendix 4 in Supplement 2). Site was represented by dummy-coded variables, with Boston as the reference category. The treatment group was dummy coded, with 1 for intervention and 0 for control. Time of assessment was coded in months and centered at the 6-month assessment, yielding codes –4 for the 2-month, –2 for the 4-month, 0 for the 6-month, and 6 for the 12-month assessments. This coding allows the dummy-coded treatment effect to reflect the contrast at 6 months.

To account for possible deviation from the linear time trend at 12 months, we included a variable coded 0 for the first 3 assessments and 6 for the final assessment. We tested whether the differences between control and intervention were attenuated over time. The hierarchical nature of multilevel models and clustered robust SEs accounted for the longitudinal data structure and nonindependence of patient outcomes nested within clinicians or clinics. Because the randomization balances observed and unobserved confounders, our main analysis controls for no further covariates. Even after adjusting for possible confounders, we observed no differences from the main results. We examined in post hoc analyses whether the treatment effect was stronger among those with moderate to severe symptoms (compared with mild) at the baseline assessment (eFigure 2 in Supplement 2) and among those who received sessions primarily by telephone (compared with in person).

We conducted additional prespecified analyses to determine whether the treatment effect on symptom reduction varied by *dose*, defined as the number of intervention sessions patients received. The dose variable was categorized as 0 (enhanced usual care control group and treatment group with no sessions), 1 to 3 sessions (minimal intervention), or 4 or more sessions (adequate intervention). Although these analyses no longer relied on randomization, they evaluated whether a greater dose was associated with stronger benefits for 6-month outcomes. Statistical significance was indicated by 2-sided *P* ≤ .05 using 2-tailed *t* tests at α = .05. Analyses were conducted in Stata, version 15 (StataCorp LLC).^21^

## Results

Overall, 341 participants enrolled across the 3 participating institutions between September 2, 2014, to May 27, 2016 (Figure).^35^ Participants were randomized to either the IIDEA treatment group (n = 172, of whom 78 [45.3%] were male and 94 [54.7%] were female, with a mean [SD] age of 33.5 [11.6] years) or the enhanced usual care control group (n = 169, of whom 89 [52.7%] were male and 80 [47.3%] were female, with a mean [SD] age of 34.3 [11.8] years). Participants originated from 17 countries (not including the United States or Spain) and had lived in the host countries from less than 1 year to more than 30 years. Of the 341 participants, 311 (91.2%) were Latino immigrants born outside of the United States or Spain. Table 1 presents the sociodemographic and clinical characteristics at baseline. No baseline differences were seen between the treatment and control groups. More than 50 participants (31.4%) in each group had positive findings from urine drug tests at baseline.

**Table 1.** Baseline Characteristics of the Intent-to-Treat Population

Variable	IIDEA Treatment Group (n = 172)	EUC Control Group (n = 169)
Characteristics, No. (%)^a^		
Study site location		
Boston, Massachusetts	44 (25.6)	37 (21.9)
Madrid, Spain	41 (23.8)	43 (25.4)
Barcelona, Spain	87 (50.6)	89 (52.7)
Age		
18-34 y	100 (58.1)	96 (56.8)
35-49 y	48 (27.9)	48 (28.4)
≥50 y	24 (14.0)	25 (14.8)
Sex		
Male	78 (45.3)	89 (52.7)
Female	94 (54.7)	80 (47.3)
Race/ethnicity		
White	29 (17.0)	31 (18.3)
Black	9 (5.3)	9 (5.3)
Indigenous/Native American	9 (5.3)	17 (10.1)
Latino/Caribbean	21 (12.3)	14 (8.3)
Mixed	103 (60.2)	98 (58.0)
Educational level		
<High school diploma	68 (39.5)	63 (37.3)
≥High school diploma, GED, or vocational school	104 (60.5)	106 (62.7)
Total personal income before tax in past year		
<US $15 000	142 (83.5)	149 (88.7)
≥US $15 000	28 (16.5)	19 (11.3)
Recruitment source		
Primary care clinic	75 (43.6)	73 (43.2)
Community-based organization	40 (23.3)	38 (22.5)
Emergency department	10 (5.8)	9 (5.3)
Patient referral	47 (27.3)	49 (29.0)
Citizenship status (United States or Spain)		
Noncitizen	78 (46.2)	69 (41.3)
Citizen	91 (53.8)	98 (58.7)
Sense of belonging		
No	70 (40.9)	69 (41.3)
Yes	101 (59.1)	98 (58.7)
Time in United States or Spain, mean (SD), y	10.03 (8.61)	10.96 (8.76)
Home country visits in past 12 mo, mean (SD), No.	0.20 (0.46)	0.31 (0.58)
Measures outcome, mean (SD)^b^		
Discrimination scale score	17.91 (8.20)	17.97 (7.75)
Ethnic identity scale score	9.44 (1.88)	9.49 (2.08)
Family conflict scale score	2.25 (2.01)	2.21 (1.91)
ASI Lite–drug score	0.04 (0.07)	0.05 (0.09)
ASI Lite–alcohol score	0.22 (0.21)	0.21 (0.17)
PHQ-9–depression score	10.88 (5.52)	10.96 (5.95)
GAD-7 score	8.53 (4.91)	8.65 (5.24)
PCL-5 score	27.19 (16.87)	25.73 (17.54)
HSCL-20 score	1.55 (0.78)	1.48 (0.82)
Mental health score^c^	38.30 (19.46)	37.99 (20.63)
Substance use score^c^	21.38 (14.26)	23.21 (14.78)
DAST-10 score	1.27 (2.14)	1.46 (2.46)
AUDIT-C score	5.20 (3.57)	5.60 (3.35)
BDEPQ score	2.13 (4.28)	2.11 (4.23)
Mindfulness score^c^	3.75 (1.09)	3.78 (1.10)
Positive urine test result, No. (%)^d^		
No	118 (68.6)	116 (68.6)
Yes	54 (31.4)	53 (31.4)
Trauma exposure, No. (%)		
No	15 (8.7)	13 (7.7)
Yes	157 (91.3)	156 (92.3)

Abbreviations: ASI, Addiction Severity Index (score range, 0-1); AUDIT, Alcohol Use Disorders Identification Test (score range, 0-12); BDEPQ, Benzodiazepine Dependence Questionnaire (score range, 0-27); DAST, Drug Abuse Screening Test (score range, 0-10); EUC, enhanced usual care; GAD, Generalized Anxiety Disorder (score range, 0-21); GED, General Educational Diploma; HSCL, Hopkins Symptom Checklist (score range, 0-4); IIDEA, Integrated Intervention for Dual Problems and Early Action; PCL, Posttraumatic Stress Disorder Checklist (score range, 0-80); PHQ, Patient Health Questionnaire (score range, 0-27).

^a^ Demographic and clinical characteristics are presented before the imputation of missing data for race/ethnicity (missing 1 observation), personal income (missing 3), citizenship status (missing 5), sense of belonging (missing 3), number of years in United States or Spain (missing 33), and number of home country visits (missing 2). The following measures are missing 2 observations each: discrimination scale (score range, 9-54, with higher scores indicating more discrimination), racial/ethnic identity scale (score range, 3-12, with higher scores indicating closer identification with others from the same culture or ethnic/racial descent), and family conflict scale (score range, 0-8, with higher scores indicating more family conflicts).

^b^ Missing observations of baseline outcomes are list-wise deleted and SDs from the available data are reported. Mean baseline responses remain almost the same with the imputed data, except that IIDEA participants have slightly higher baseline HSCL-20 scores than the EUC participants in the imputed data. For all outcomes of substance use and mental health measures, higher scores indicate more symptoms and higher severity level.

^c^ Mental health and substance use score range: 0 to 100; mindfulness score range: 1 to 6.

^d^ Positive urine test result shows a binary outcome of drug metabolite and use of any of the 6 drug types (amphetamine, benzodiazepine, cocaine, methamphetamine, heroin, and marijuana), but it does not show alcohol misuse.

Of the 172 individuals enrolled in IIDEA, 89 (51.7%) attended more than 6 sessions (covering the core elements), 44 (25.6%) attended 1 to 5 sessions, and 39 (22.7%) did not initiate treatment (0 sessions). Participants aged 35 years or older; with a high school diploma, General Educational Development, or higher educational level; and with no children were substantially more likely to complete treatment. Compared with participants who did not complete treatment at baseline, those who completed had statistically significantly higher mental health symptoms on the HSCL-20 (*t* = 3.03; *P* = .003) and the Benzodiazepine Dependence Questionnaire (*t* = 2.85; *P* = .005).

Table 2 presents the results of the intent-to-treat analysis for primary and secondary outcomes. With regard to the primary outcomes, no statistically significant treatment effects on substance use were found at the 6-month follow-up (ASI Lite–drug score: β = −0.02 [SE, 0.69; *P* = .88; Cohen *d*, 0.00; 95% CI, −0.17 to 0.17]; ASI Lite–alcohol score: β = −0.01 [SE, 1.19; *P* = .66; Cohen *d,* 0.00; 95% CI, −0.12 to 0.12]; urine drug test result: β = −0.36 [SE, 0.43; *P* = .50; OR, 0.70; 95% CI, 0.30-1.61]). No adverse effects were identified in any intervention cases. However, the analysis of secondary outcomes showed that IIDEA was effective in decreasing depressive symptoms per the PHQ-9 score (β = −1.14; SE, 0.47; *P* = .02; Cohen *d*, 0.20; 95% CI, 0.04-0.36), PTSD symptoms per the PCL-5 score (β = −3.23; SE, 1.59; *P* = .04; Cohen *d*, 0.25; 95% CI, 0.01-0.37), and overall mental health symptoms per the HSCL-20 (β = −0.20; SE, 0.07; *P* = .01; Cohen *d*, 0.25; 95% CI, 0.08-0.42) and composite mental health (β = −3.70; SE, 1.75; *P* = .04; Cohen *d*, 0.19; 95% CI, 0.01-0.36) scores at the 6-month follow-up.

**Table 2.** Primary and Secondary Outcomes Evaluated at 6-Month Follow-up of the Intent-to-Treat Population

Characteristic	β (SE)								
	ASI Lite–Drug Score ^a^	ASI Lite–Alcohol Score ^a^	Positive Urine Test Result^b^	PHQ-9 Score	GAD-7 Score	PCL-5 Score	HSCL-20 Score	Mental Health Score	Substance Use Score
IIDEA	−0.02 (0.69)	−0.01 (1.19)	−0.36 (0.43)	−1.14 (0.47)^c^	−0.61 (0.45)	−3.23 (1.59) ^c^	−0.20 (0.07)^d^	−3.70 (1.75) ^c^	−1.27 (1.09)
Time^e^	−0.10 (0.16)	−1.00 (0.34)^d^	0.02 (0.08)	−0.40 (0.11)^f^	−0.46 (0.09)^f^	−1.45 (0.31)^f^	−0.07 (0.01)^f^	−1.82 (0.36)^f^	−0.54 (0.26)^c^
IIDEA × time	0.10 (0.17)	0.51 (0.38)	−0.06 (0.12)	−0.03 (0.15)	0.14 (0.12)	0.55 (0.48)	−0.00 (0.02)	0.41 (0.52)	0.08 (0.38)
(Time – *t*)^g^	0.07 (0.21)	1.01 (0.43)^c^	−0.05 (0.11)	0.30 (0.16)	0.47 (0.13)^f^	1.15 (0.40)^d^	0.06 (0.02)^d^	1.60 (0.50)^d^	0.55 (0.33)
IIDEA × (time – *t*)	−0.11 (0.26)	−0.56 (0.51)	0.09 (0.18)	0.18 (0.20)	−0.16 (0.17)	−0.27 (0.61)	0.01 (0.03)	−0.14 (0.68)	−0.18 (0.52)
Madrid, Spain sites^h^	−0.28 (0.77)	0.41 (1.65)	−0.44 (0.33)	0.02 (0.43)	−0.25 (0.46)	−2.60 (1.27) ^c^	−0.05 (0.08)	−1.28 (1.62)	2.47 (1.29)
Barcelona, Spain sites	−0.17 (0.60)	−0.38 (0.81)	−0.25 (0.34)	−0.53 (0.39)	−0.91 (0.38)^c^	−4.11 (1.11)^f^	−0.13 (0.06)^c^	−3.14 (1.39)^c^	2.02 (0.88)^c^
Baseline outcome	0.57 (0.04)^f^	0.47 (0.04)^f^	2.86 (0.30)^f^	0.53 (0.04)^f^	0.49 (0.04)^f^	0.55 (0.04)^f^	0.49 (0.04)^f^	0.59 (0.04)^f^	0.51 (0.04)^f^
Constant	1.95 (0.78)^c^	6.89 (1.44)^f^	−1.85 (0.38)^f^	2.66 (0.52)^f^	2.62 (0.49)^f^	8.92 (1.63)^f^	0.44 (0.08)^f^	8.46 (2.04)^f^	5.57 (1.27)^f^

Abbreviations: ASI, Addiction Severity Index (score range, 0-1); GAD, Generalized Anxiety Disorder (score range, 0-21); HSCL, Hopkins Symptom Checklist (score range, 0-4); IIDEA, Integrated Intervention for Dual Problems and Early Action; PCL, Posttraumatic Stress Disorder Checklist (score range, 0-80); PHQ, Patient Health Questionnaire (score range, 0-27).

^a^ ASI Lite–drug and ASI Lite–alcohol scores range from 0 to 1, but participant scores on these measures were rescaled to a range of 0 to 100 before regression analyses (ie, multiplied by 100). This adjustment was made to ensure meaningful regression estimates.

^b^ Positive urine test result shows a binary outcome of drug metabolite and use of any of the 6 drug types (amphetamine, benzodiazepine, cocaine, methamphetamine, heroin, and marijuana), but it does not show alcohol misuse. Coefficients in terms of the log odds are reported for binary outcome.

^c^ *P* < .05.

^d^ *P* < .01.

^e^ Time is a continuous variable that equals to –4 for research assessment 2, –2 for research assessment 2, 0 for research assessment 3, and 6 for research assessment 5.

^f^ *P* < .001.

^g^ Time – *t* is a continuous variable that equals to 6 for research assessment 5 and 0 otherwise; *t* denotes the time when treatment ends, which equals to 6.

^h^ The reference group comprises patients in the enhanced usual care control group, and the reference site is Boston, Massachusetts.

Table 3 reports results of exploratory analyses of whether intervention effects were moderated by baseline symptom severity. For those with moderate to severe baseline substance use and mental health symptoms, IIDEA was effective in reducing substance use per the urine test result (OR, 0.25; 95% CI, 0.09-0.67; *P* = .01), depression per the PHQ-9 score (Cohen *d,* 0.38; 95% CI, 0.15-0.62; *P* = .001), anxiety per the GAD-7 score (Cohen *d*, 0.34; 95% CI, 0.06-0.63; *P* = .02), PTSD per the PCL-5 score (Cohen *d,* 0.37; 95% CI, 0.08-0.65; *P* = .01), and overall mental health (Cohen *d,* 0.44; 95% CI, 0.21-0.67; *P* < .001) symptoms, when compared with the enhanced usual care control counterparts. The treatment effects on substance use and the urine test results at 6 months were statistically significantly larger for those with moderate to severe baseline symptoms than those with mild symptoms (ASI Lite—drug score: β = −4.71 [SE, 2.25; *P* = .04; Cohen *d,* 0.59; 95% CI, 0.04-1.13]; urine test result: β = −1.64 [SE, 0.47; *P* < .001; OR, −1.64; 95% CI, 0.07-0.49]). With regard to secondary outcomes, reduction in mental health symptoms was also greater among participants with moderate to severe mental health symptoms (PHQ-9 score: β = −2.28 [SE, 0.72; *P* = .001; Cohen *d,* 0.40; 95% CI, 0.15-0.64]; GAD-7 score: β = −1.72 [SE, 0.78; *P* = .03; Cohen *d,* 0.34; 95% CI, 0.04-0.64]; PCL-5 score: β = −5.35 [SE, 2.71; *P* = .048; Cohen *d,* 0.31; 95% CI, 0.01-0.64]; HSCL-20 score: β = −0.31 [SE, 0.10; *P* = .003; Cohen *d,* 0.39; 95% CI, 0.14-0.63]), in contrast to those with mild symptoms.

**Table 3.** Primary and Secondary Outcomes by Baseline Severity Level Evaluated at 6-Month Follow-up of the Intent-to-Treat Population

Characteristic	β (SE)								
	ASI Lite–Drug Score	ASI Lite–Alcohol Score	Positive Urine Test Result	PHQ-9 Score	GAD-7 Score	PCL-5 Score	HSCL-20 Score	Mental Health Score	Substance Use Score
IIDEA reference^a^	0.53 (0.46)	−0.22 (1.51)	0.25 (0.45)	0.10 (0.51)	−0.00 (0.50)	−0.93 (1.72)	−0.05 (0.08)	0.90 (1.86)	−0.83 (1.22)
IIDEA moderate to severe at baseline^b^	−4.71 (2.25)^c^	2.27 (1.95)	−1.64 (0.47)^d^	−2.28 (0.72)^e^	−1.72 (0.78)^c^	−5.35 (2.71)^c^	−0.31 (0.10)^e^	−9.79 (2.97)^d^	−1.48 (2.08)
Moderate to severe at baseline	13.20 (1.58)^d^	10.22 (1.89)^d^	3.70 (0.38)^d^	6.18 (0.50)^d^	5.12 (0.50)^d^	18.84 (1.83)^d^	0.73 (0.09)^d^	23.31 (2.07)^d^	11.99 (1.61)^d^
Time	−0.10 (0.16)	−1.00 (0.34)^e^	0.02 (0.08)	−0.40 (0.11)^d^	−0.46 (0.09)^d^	−1.45 (0.31)^d^	−0.07 (0.01)^d^	−1.82 (0.36)^d^	−0.54 (0.26)^c^
IIDEA × time	0.10 (0.17)	0.51 (0.38)	−0.06 (0.12)	−0.03 (0.15)	0.14 (0.12)	0.55 (0.48)	−0.00 (0.02)	0.41 (0.52)	0.08 (0.38)
(Time − *t*)	0.07 (0.21)	1.01 (0.43)^c^	−0.05 (0.12)	0.30 (0.16)	0.47 (0.13)^d^	1.15 (0.40)^e^	0.06 (0.02)^e^	1.60 (0.50)^e^	0.55 (0.33)
IIDEA × (time − *t*)	−0.11 (0.26)	−0.56 (0.51)	0.09 (0.18)	0.18 (0.20)	−0.16 (0.17)	−0.27 (0.61)	0.01 (0.03)	−0.14 (0.68)	−0.18 (0.52)
Madrid, Spain sites	−0.39 (0.97)	−0.58 (2.68)	−0.39 (0.32)	0.17 (0.47)	−0.26 (0.51)	−1.89 (1.45)	−0.02 (0.10)	−0.36 (1.97)	1.85 (1.81)
Barcelona, Spain sites	−0.75 (0.67)	−2.93 (1.46)^c^	−0.20 (0.34)	−1.00 (0.39)^c^	−1.25 (0.39)^e^	−6.11 (1.31)^d^	−0.18 (0.07)^c^	−5.50 (1.59)^d^	1.40 (1.23)
Constant	2.89 (0.71)^d^	11.36 (1.63)^d^	−2.19 (0.40)^d^	5.32 (0.42)^d^	5.13 (0.38)^d^	17.60 (1.40)^d^	0.85 (0.09)^d^	19.70 (1.65)^d^	11.36 (1.18)^d^
Effect size (95% CI)^f^	0.52 (−0.06 to 1.1)	0.10 (−0.05 to 0.26)	0.25 (0.09 to 0.67)^e^	0.38 (0.15 to 0.62)^e^	0.34 (0.06 to 0.63)^c^	0.37 (0.08 to 0.65)^c^	0.44 (0.21 to 0.67)^d^	0.44 (0.18 to 0.71)^e^	0.16 (−0.10 to 0.41)

Abbreviations: ASI, Addiction Severity Index (score range, 0-1); GAD, Generalized Anxiety Disorder (score range, 0-21); HSCL, Hopkins Symptom Checklist (score range, 0-4); IIDEA, Integrated Intervention for Dual Problems and Early Action; PCL, Posttraumatic Stress Disorder Checklist (score range, 0-80); PHQ, Patient Health Questionnaire (score range, 0-27).

^a^ The reference is mild symptoms at baseline.

^b^ Moderate to severe symptoms at baseline is a dummy-coded indicator that equals to 1 if baseline severity equals or exceeds the moderate symptoms of the outcome and to 0 if otherwise (eAppendix 4 in Supplement 2).

^c^ *P* < .05.

^d^ *P* < .001.

^e^ *P* < .05.

^f^ Effect sizes were calculated in the metric of odds ratio for binary outcomes and Cohen *d* for continuous outcomes; approximate 95% CIs are reported here. For participants with moderate to severe symptoms at baseline, the treatment effect size was derived from the model estimates for each outcome.

Table 4 shows that an adequate dose (>4 sessions) was associated with decreasing drug use (β = −1.37; SE, 0.67; *P* = .048; Cohen *d,* 0.17; 95% CI, 0.01- 0.33) and lowering the substance use composite score (β = −4.04; SE, 1.19; *P* = .002; Cohen *d,* 0.28; 95% CI, 0.12-0.44). Compared with those who received fewer than 4 sessions, participants who received 4 or more sessions also improved their depressive symptoms per the PHQ-9 score (β = −1.70; SE, 0.65; *P* = .01; Cohen *d,* 0.30; 95% CI, 0.07-0.52), anxiety per the GAD-7 score (β = −1.27; SE, 0.58; *P* = .04; Cohen *d,* 0.25; 95% CI, 0.03-0.48), and PTSD per the PCL-5 score (β = −4.90; SE, 2.11; *P* = .03; Cohen *d,* 0.29; 95% CI, 0.04-0.53) as well as their HSCL-20 (β = −0.30; SE, 0.10; *P* = .01; Cohen *d,* 0.37; 95% CI, 0.13-0.62) and composite mental health (β = −6.38; SE, 2.29; *P* = .01; Cohen *d,* 0.32; 95% CI, 0.09-0.54) scores. A sensitivity analysis suggested larger treatment effects for those who received 6 or more sessions (eTable 2 in Supplement 2).

**Table 4.** Participation in IIDEA Treatment Evaluated at 6-Month Follow-up^a^

Characteristic	β (SE)								
	ASI Lite–Drug Score	ASI Lite–Alcohol Score	Positive Urine Test Result	PHQ-9 Score	GAD-7 Score	PCL-5 Score	HSCL-20 Score	Mental Health Score	Substance Use Score
IIDEA patients with 0-3 sessions	1.44 (0.89)	2.74 (1.70)	−0.19 (0.45)	−0.70 (0.69)	0.55 (0.77)	−0.80 (2.10)	−0.01 (0.09)	0.14 (2.80)	3.47 (2.09)
IIDEA patients with ≥4 sessions	−1.37 (0.67)^b^	−0.89 (1.38)	−0.60 (0.52)	−1.70 (0.65)^b^	−1.27 (0.58)^b^	−4.90 (2.11)^b^	−0.30 (0.10)^c^	−6.38 (2.29)^c^	−4.04 (1.19)^c^
Madrid, Spain sites^d^	−1.27 (1.12)	−1.14 (2.21)	−0.40 (0.60)	−0.24 (0.71)	−0.45 (0.70)	−2.34 (1.72)	−0.02 (0.13)	−1.97 (2.32)	1.39 (1.66)
Barcelona, Spain sites	−1.39 (0.82)	−0.28 (1.32)	−0.53 (0.36)	−1.52 (0.65)^b^	−1.50 (0.39)^e^	−5.73 (1.22)^e^	−0.18 (0.10)	−6.13 (1.62)^e^	0.12 (1.09)
Baseline outcome	0.61 (0.08)^e^	0.44 (0.05)^e^	2.28 (0.36)^e^	0.47 (0.05)^e^	0.46 (0.07)^e^	0.51 (0.06)^e^	0.49 (0.07)^e^	0.54 (0.06)^e^	0.54 (0.06)^e^
Intercept	1.77 (0.94)	6.39 (1.82)^c^	−1.49 (0.33)^e^	3.64 (0.85)^e^	2.70 (0.73)^e^	9.29 (1.72)^e^	0.41 (0.14)^c^	10.18 (2.99)^c^	4.19 (1.79)^b^
Sample size, No.^f^	268	268	257	268	268	268	216	268	268
Effect size (95% CI)^g^	0.17 (0.01 to 0.33)	0.04 (−0.09 to 0.18)	0.60 (−0.42 to 1.62)	0.30 (0.07 to 0.52)	0.25 (0.03 to 0.48)	0.29 (0.04 to 0.53)	0.37 (0.13 to 0.62)	0.32 (0.09 to 0.54)	0.28 (0.12 to 0.44)

Abbreviations: ASI, Addiction Severity Index (score range, 0-1); GAD, Generalized Anxiety Disorder (score range, 0-21); HSCL, Hopkins Symptom Checklist (score range, 0-4); IIDEA, Integrated Intervention for Dual Problems and Early Action; PCL, Posttraumatic Stress Disorder Checklist (score range, 0-80); PHQ, Patient Health Questionnaire (score range, 0-27).

^a^ Missing data are list-wise deleted. The analytical sample for each regression slightly varies because the missing data differ for each outcome.

^b^ *P* < .05.

^c^ *P* < .01.

^d^ The reference group comprises patients in the enhanced usual care control group, and the reference site is Boston, Massachusetts.

^e^ *P* < .001.

^f^ Sample size varies because of missing data in a specific estimation.

^g^ Effect sizes were calculated in the metric of odds ratio for binary outcomes and Cohen *d* for continuous outcomes. Odds ratios were calculated as an exponent of the regression coefficient from the estimated logit model. Approximate 95% CIs are reported here, assuming the regression coefficients are normally distributed approximately.

Compared with Boston patients, Barcelona patients had a statistically significantly lower GAD-7 score (*t* = −2.41; *P* = .02), PCL-5 score (*t* = −3.69; *P* < .001), and HSCL-20 score (*t* = −1.99; *P* = .047) at 6-month follow-up after adjusting for baseline severity, whereas Madrid patients had similar scores to those of Boston patients, except for PCL-5 score (*t* = −2.04; *P* = .04). Six-months after baseline, Madrid and Barcelona patients were like those in Boston in substance use severity and urine test results. No evidence of differential effectiveness by site was found, except for PHQ-9 score, in which the intervention effect appeared to be smaller in the Madrid patients (eTable 3 in Supplement 2) than in the Boston patients. The negative time coefficients suggest a decline in the mental health symptoms and alcohol use up to the 6-month follow-up for both IIDEA and enhanced usual care groups over time.

Most IIDEA patients received the treatment in person (98 [73.7%] in person vs 35 [26.3%] by telephone). In-person patients were more likely to experience improvement (eTable 4 in Supplement 2). These results are robust for alternative modeling strategies, estimation methods, and how missing data were handled (eAppendix 4 in Supplement 2). In addition, the results remain significant after Benjamini-Hochberg adjustment, assuming a false discovery rate of 0.15.

## Discussion

We evaluated IIDEA, a culturally tailored intervention that integrates the treatment of mental health symptoms and substance misuse for Latino immigrants with some level of co-occurring symptoms. No IIDEA effect on the primary alcohol and drug outcomes was found in the treatment group, but the secondary and exploratory results were shown to be effective for mental health outcomes. Intent-to-treat analyses documented IIDEA’s effectiveness for mental health outcomes, which were considered secondary in this trial but are clinically important. With regard to the primary alcohol and drug symptoms, evidence indicated that IIDEA was not effective in persons who presented with mild levels of co-occurring symptoms. Auxiliary analyses suggested that the baseline severity level and treatment dose were associated with substantial treatment effects, including on substance misuse.

Similar to previous cognitive behavioral therapy trials, this trial found a reduction in mental health symptoms, with small to moderate effect sizes.^36,37^ Participants with mild symptoms were overrepresented in this trial, as it was designed to address substance use and mental health symptoms in nondiagnosed participants who were not seeking treatment. However, this distribution of the sample may mask a moderate treatment effect for participants with moderate to severe clinical symptoms. Those with moderate to severe symptoms may benefit most from the IIDEA intervention. Telephone calls to control patients to assess their symptoms may have also had an effect by activating patients to deal with their symptoms^38^ and consequently decreasing the difference between the treatment and control groups. Relatedly, 19 control patients (11.2%) reported accessing mental health services in 1 of the follow-up interviews. In an earlier stage of the trial, we found that 12% of participants screened positive for moderate or severe co-occurring symptoms on the AC-OK screener, suggesting that a clinically significant number of people could benefit from IIDEA.

The IIDEA therapy requires 50 hours of training for clinicians to achieve competency, plus ongoing supervision, suggesting that adaptations may be needed for low-resource environments. However, adoption of the intervention by clinicians, once trained, was described anecdotally as high. For instance, an adaptation of IIDEA is being used in Spain in a project for treating pregnant women with substance use symptoms.

Engaging individuals with co-occurring symptoms in behavioral health care can be a challenge. However, IIDEA therapists were successful in initiating treatment with 77.3% of the intervention participants, who were not seeking care. More than half completed treatment, demonstrating good acceptability. This rate is higher than in other studies.^39,40^ In line with previous findings, studies show a lower probability of premature treatment termination when Latino individuals are ethnically and linguistically matched with their therapist^41^ and when interventions are culturally adapted.^42^

Understanding co-occurring symptoms as a chronic disease may help inform the conceptualization of treatments as requiring ongoing maintenance. A higher number of sessions was associated with a statistically significant effect size for reducing alcohol outcomes and a statistically significantly higher effect size for reducing depressive symptoms.^40^ The IIDEA intervention could also benefit from a maintenance component, to sustain clinical improvement and prevent relapse.^43^

Moderation analysis shows greater effectiveness among a low-income, recent immigrant population, a group that usually receives few resources.^44^ For Latino individuals with co-occurring symptoms, treatment in person appears more effective than treatment by telephone, likely because of the difficulty of maintaining attention to mindfulness exercises and cognitive restructuring.^45,46,47^ A first in-person visit may be necessary to reduce cultural mistrust and establish rapport.^45^ Finally, the sustainability of IIDEA should be further assessed, including barriers to implementation in community sites that lack institutional funding and access to continued supervision.^48^

### Strengths and Limitations 

The study has several strengths, including a diverse group of Latino immigrants from 17 countries and with varied characteristics and symptom levels. Scientific rigor was maintained, as 77% of patients completed the 12-month follow-up and 75% completed at least 3 follow-up assessments. Study results suggest that the IIDEA program is an acceptable and effective behavioral health treatment for Latino immigrants with co-occurring symptoms whose symptoms were within the moderate-to-severe range.

The study also has several limitations. We did not conduct biological screens for alcohol because of cost constraints, which diminished our ability to detect differences across groups. Disclosure concerns could have also undermined drug assessments at baseline. Participants were more willing to disclose their substance use symptoms to a trusted clinician over time.^49^

## Conclusions

Although the IIDEA program did not change drug misuse in a heterogeneous sample, it did improve secondary mental health outcomes. This treatment may provide a path for treating Latino immigrants with co-occurring symptoms with elevated or moderate-to-severe symptoms.

## Notes

Supplement 1. **Trial Protocol**

Supplement 2. **eAppendix 1.** Study Design of the IIDEA RCT **eAppendix 2.** Description of the Intervention and Fidelity **eAppendix 3.** Analytical Methods—Data Preparation **eAppendix 4.** Analytical Methods—Data Analysis **eFigure 1.** IIDEA Study Flow **eFigure 2.** Heterogenous Treatment Effects on Substance Misuse Outcomes Across Different Subgroups **eTable 1.** List of Assessment Measures/Variables **eTable 2.** Analysis of Participation Rates (Receiving 6+ Sessions) in IIDEA Intervention on Main Outcomes at 6-Month Follow-up **eTable 3.** Intent-to-Treat Analysis of IIDEA Intervention on Main Outcomes by Site **eTable 4.** Intent-to-Treat Analysis of IIDEA Intervention on Main Outcomes by Received Intervention Type **eTable 5.** Significant Baseline Differences Between Participants With Missing/Incomplete Assessments and Participants With Complete Assessments

Supplement 3. **Data Sharing Statement**
