# Supplementary material for: Evaluation of the Integrated Intervention for Dual Problems and Early Action Among Latino Immigrants With Co-occurring Mental Health and Substance Misuse Symptoms: A Randomized Clinical Trial
Source: JAMA Netw Open. 2019 Jan 11;2(1):e186927. doi: 10.1001/jamanetworkopen.2018.6927 (PMC6484537; doi:10.1001/jamanetworkopen.2018.6927)
Supplement: Supplement 1. — Trial Protocol [file jamanetwopen-2-e186927-s001.pdf]

Supplementary Online Content

Alegría M, Falgas-Bague I, Collazos F, et al. Evaluation of the Integrated Intervention for Dual Problems and Early Action among Latino immigrants with co-occurring mental health and substance misuse symptoms: a randomized clinical trial. *JAMA Netw Open.* 2019;2(1):e186927. doi:10.1001/jamanetworkopen.2018.6927

eAppendix 1. Study Design of the IIDEA RCT

eAppendix 2. Description of the Intervention and Fidelity

eAppendix 3. Analytical Methods—Data Preparation

eAppendix 4. Analytical Methods—Data Analysis

eFigure 1. IIDEA Study Flow

eFigure 2. Heterogenous Treatment Effects on Substance Misuse Outcomes Across Different Subgroups

eTable 1. List of Assessment Measures/Variables

eTable 2. Analysis of Participation Rates (Receiving 6+ Sessions) in IIDEA Intervention on Main Outcomes at 6-Month Follow-up

eTable 3. Intent-to-Treat Analysis of IIDEA Intervention on Main Outcomes by Site

eTable 4. Intent-to-Treat Analysis of IIDEA Intervention on Main Outcomes by Received Intervention Type

eTable 5. Statistically Significant Baseline Differences Between Participants With Missing/Incomplete Assessments and Participants With Complete Assessments

This supplementary material has been provided by the authors to give readers additional information about their work.

© 2019 Alegría et al. *JAMA Network Open.*

eAppendix 1. Study Design of the IIDEA RCT

STUDY DESIGN SUMMARY

We conducted the IIDEA randomized clinical trial between September 2014 and February 2017, with mostly Latino immigrants in Massachusetts and at two sites in Spain. Based on the promising results of the AC-OK as a screener for dual diagnosis in this population[1], we used the AC-OK as the screening tool for the clinical trial. Eligible participants needed to endorse two mental health items and two substance use items on the AC-OK, and not be in specialty behavioral healthcare in the past 3 months or have an appointment planned. The trial design included five research assessments: baseline, and follow up assessment at two months, four-months, six-months, and twelve-months from baseline. Participants were also administered a Computerized Adaptive Testing interview for mental health (CAT-MH), funded by a research supplement (NIMH; PI: Gibbons). Participants were randomized into either a 10-session intervention or a control group that received regular check-ins with a care manager. Participants were also provided access to HIV and STI testing by the study team and urine testing for drug metabolites as part of administrative supplement to the NIDA grant (PI: Alegria).

STUDY SETTING

Participants for the randomized controlled trial were identified through recruitment in primary care clinics, community sites, and emergency departments, as well as from referrals from patients to others in their social network who might benefit from the program. Recruitment was conducted between September 2014 and May 2016 in Boston, Madrid and Barcelona. Approval was obtained from the institutional review boards of participating institutions. We held a series of presentations with Directors and staff in the clinics and community sites to introduce the study and determine optimal procedures for outreach and recruitment of patients.

SAMPLING, RECRUITMENT AND CONSENT

Research Assistants (RAs) approached potential participants in person in the waiting room of clinics and community agencies. In certain sites, RAs took contact information for patients and followed up by phone to either screen or administer informed consent, depending on the permission granted by each institutional review board. In some cases, RAs called a list of patients made available by clinical referral, directors of the site, or by access to electronic data.

Research staff first obtained informed consent from participants and then utilized the study screening to identify patients who were eligible for the study using the AC-OK short screener. We asked participants' permission to contact them through either their PCP or two close contacts that had had the most frequent or stable contact with the participant over the past six months. We collected an address to send intervention materials and follow up letters and establish contact with the participant.

Patients who were NOT eligible received only the short screener and the CAT-MH interview and were compensated $20. The CAT-MH™ is a brief computerized adaptive mental health screening that was validated in Spanish with this sample as part of a NIMH research supplement[2,3].

Patients who were eligible received the short screener and were scheduled for a follow up baseline interview. The baseline interview included instruments designed to identify mental health problems, substance misuse and HIV risk behaviors, as well as socio-demographics, cultural, contextual and social factors, medication use, chronic conditions, disability in daily activities, health literacy, language proficiency, access to health services, migration history, and assessments of illness management/recovery and mindfulness. At baseline, they were also administered the CAT-MH interview and a urine test for drug metabolites to determine drug use. They were administered a capacity to consent form to ensure they understood and could take part in the full trial. They were compensated $40-50 for the time spent answering the assessment, depending on what assessment they were completing (see below). Upon completion of baseline, participants were randomized into either the intervention or control condition and began the trial. Additional interviews were administered by research assistants blind to study condition at 2 months following baseline, 4 months, 6 months, and 12 months. At each interview, an urine test was administered for drug metabolites.

Emergency protocol: We used an emergency protocol throughout the screening phase and in the trial. If participants responded affirmatively to questions in the screener or interview related to suicidal thoughts, they were administered the Paykel suicidality screener[5] after the interview. The emergency procedure was prompted if a patient endorsed a 4

or 5 on the Paykel suicidality screener over the past 30-days. The questions include: "Has there been a time in the last 30 days when you reached the point where you seriously considered taking your own life, or perhaps made plans how you should go about doing it?" = yes or "Has there been a time in the past 30 days/since the last interview when you made an attempt on your own life?" = yes. In Massachusetts, study staff connected the patient by phone or in person to the BEST emergency services team, which performs an assessment of patient safety and provides referrals in the event immediate care is needed. In the two sites in Spain, participants were referred to clinical staff overseeing the study, who assessed and helped connect with emergency care if needed. Participants with active suicidality could be contacted after 30 days for reassessment to see if they could safely participate in the trial, if they were not actively receiving psychotherapy.

Certificate of Confidentiality: We received a Certificate of Confidentiality from NIDA for the US-based participant in this project.

RESEARCH PROCEDURES

We recruited bilingual and bicultural study staff who would be engaging, non-judgmental, persistent, and not easily discouraged. Given the sensitivity around substance use and mental health issues and potential concerns about immigration status and discrimination, we selected bilingual Spanish/English staff, who were also Latino and were sometimes born outside of the US or Spain, to build trust and familiarity with participants. All RAs received a comprehensive training to ensure they could conduct the interview effectively and make the participant feel comfortable. RAs followed 4 steps as part of their training 1) review of assessment questionnaire and materials, 2) remote training via WebEx, 3) conduct of at least two successful role plays of the interviews; and 4) intense quality control review of the audio recording of first 2 patient interviews, including receiving detailed feedback from a study supervisor. During the study, all interviews were recorded and periodically evaluated under quality control procedures. A minimum of 15% of all the interviews in the study were randomly selected, distributed to the quality control team and evaluated by the project coordinator of each site for feedback to the interviewer, and corrections if necessary.

eAppendix 2. Description of the IIDEA Intervention and Fidelity

The Integrated Intervention for Dual Problems and Early Action (IIDEA) is a 10-12 session, transdiagnostic, manualized therapy. It was developed to address elevated symptoms of mental health (depression, anxiety, trauma) and substance use (drug, alcohol, and benzodiazepine misuse) in Latino adults.

IIDEA uses a transdiagnostic model, based in research showing that Latino immigrants have a range of mild to moderate co-occurring behavioral symptoms, but low rates of mental health services use and treatment engagement[4-8]. The intervention employs evidence-based components shown effective in transdiagnostic applications (such as Cognitive Behavioral Therapy; CBT). The intervention seeks to improve engagement among Latinos by addressing cultural and psychosocial barriers through offering sessions in the language of the participant, either in-person or by phone, and scheduling sessions at the convenience of the patient. Additionally, it incorporates mindfulness practice to improve awareness of thoughts and reactions to stress, together with CBT and strategies for emotional regulation[9-12]. Finally, it includes components for HIV/STI prevention by offering HIV/STI counseling and testing and discussing common myths about these illnesses.

The intervention incorporates information from previous manuals that have demonstrated efficacy in different components. For instance, along with co-authors Fortuna and Mueser, we adapted and expanded components of Mindfulness Based Cognitive Therapy for the Treatment of PTSD and SUD in Adolescents (MBCT-DUAL)[13] and CBT for PTSD[14]. These manuals demonstrated efficacy in improving PTSD, anxiety, depression symptoms and substance use severity[13,15]. To address HIV prevention, we selected components of two interventions proven to be effective in decreasing HIV risk: CONNECT[16-18] and RESPECT[19-21], and motivation interviewing techniques were incorporated throughout the intervention[22]. The IIDEA program includes a participant's manual containing information presented during the session and exercises to practice during the week.

The program is structured in 10-12 sessions that are expected to take one hour per session, administrated weekly within a maximum period of 6 months. The first session emphasizes engaging the participant through motivational interviewing, cultural formulation and assessment of barriers to care. It also provides an overview of the program and psychoeducation on the identified symptoms. In the second session, the clinician introduces the concept of mindfulness and its practice as well as the principles of CBT, including addressing patterns of negative thoughts, and their relationship with feelings and actions. In the third session, the 5 steps of cognitive restructuring are presented and practiced, and this work continues throughout the 3 subsequent sessions and supplemental activities. Fourth, fifth and sixth sessions emphasize drug use psychoeducation, relapse prevention techniques and effective communication. Sessions seven and eight work on HIV and STI risk behavior prevention, including pre- and post-test counseling and testing for HIV, chlamydia and gonorrhea. Finally, the ninth and tenth session focus on reviewing techniques and building a self-care plan. Clinicians also offer referrals for continued therapy for patients who need it or request ongoing care.

The intervention is delivered in-person or by phone using participant's preferred language (English or Spanish). In cases where the participants have childcare or illness constraints, home visits are offered.

Through an iterative process, a fidelity rating instrument was developed to include items common across all sessions and specific to the skill sets for each session. The instrument consisted of a quantitative section that measured the completion of the session components and a qualitative section that rated the quality of session delivery by the clinician. The first section scored each component with 2 (indicating that the clinician addressed the component fully), 1 (indicating partial fulfilment), or 0 (indicating that the clinician did not address the component at all). The qualitative section of the fidelity scale included four questions concerning the quality of therapy administration, which applied to all sessions. These covered: Patient understanding, clinician empathy with the patient, time management and overall ability to deliver the key components of the session. These items were scored out of 3, and were given the following rating instructions: 0 = Not at all (the clinician does not carry out any of the skills required; the clinician does not cover any of the important themes of the session); 1= Poor (the patient does not understand most of the important themes); 2=Acceptable (the patient does not understand all of the important themes); 3 = Good (The clinician fulfills all of the requirements. Although there could be some global criticisms, all the elements were reasonably well covered, and the patient understands and can apply the contents).

© 2019 Alegría et al. *JAMA Network Open.*

eAppendix 3. Analytical methods – data preparation

Some variables had missing data due to drop-outs, missing assessments, lack of response to questions, or participant assessment of a question as not applicable. To account for missing data, we used multiple imputation methods in Stata version 14.2 via the *mi impute chained* command[23]. The multiple imputations were carried out in three steps: first, we imputed the missing data for all the variable considered, creating 20 imputed datasets that each consist of 341 participants with four follow-up assessments per participant. Second, we ran analysis on each individually imputed data set; third, we aggregated the individual estimate to obtain final estimates and adjusted standard errors for the uncertainty due to imputation[24].

Each imputation was carried out using the chained equations method[25]. In what follows, we refer to variables with missing data as incomplete variables and those with non-missing data as complete variables. Each incomplete variable x_j was specified as a conditional function (g_j) given the set of all other variables used in the imputation, comprising both incomplete ($x_1,\ldots,x_m$) and complete variables (Z). We fitted the conditional model g_j to generate the predicted values of x_j using an iterative method. Specifically, each incomplete variable x_j was iteratively estimated and in each iteration the variable x_j^t was then updated to x_j^{t+1} based on the conditional model. This updated variable was then used in the estimations of the other variables, following the conditional model specification: $x_j^{t+1} \sim g_j (x_j \mid x_j^{t+1},\ldots, x_{j-1}^{t+1}, x_{j+1}^{t},\ldots Z, \phi_j)$, for $j\in\{1..m\}$, where ϕ_j j were parameters of the conditional model g_j . These steps were repeated for all variables $x_1,\ldots, x_m$ and, after an initial burn-in phase, the procedure was stopped once convergence was reached.

The variables used for imputation included main outcome variables and their baseline measures, patient socio-demographics, clinical characteristics, and study design variables, such as site and intervention indicators, as well as dummy indicators for each follow-up assessment.

We used interval regressions to incorporate the theoretical bounds of the clinical outcome variables to increase the efficiency and accuracy of the imputation procedure, (e.g., we restricted the imputed values of PHQ-9 to lie within the 0 to 27 intervals). To impute the binary variables, e.g. whether the urine drug test was positive, logistic regressions were used. Other conditional models were specified to be multiple linear regressions.

After running analysis on the twenty data sets we imputed via step 1, we combined these separate estimates from the different data sets to arrive at our final estimates.

© 2019 Alegría et al. *JAMA Network Open.*

eAppendix 4. Analytical methods – data analysis

First, we compared distributions of baseline characteristics between participants who received the IIDEA intervention and participants in the usual care group, to assess the balance of the observed covariates. For each follow-up assessment, we compared the baseline covariates to detect any statistically significant baseline differences between those who completed assessment versus those who did not.

Second, to assess the effect of the intervention, we conducted an intent-to-treat (ITT) analysis, using a multilevel, multivariate regression model to assess changes in outcome variables over time in the treatment and control groups. To account for the nature of longitudinal data, the ITT analysis was carried out by fitting multilevel mixed-effects models to allow for valid variance calculation and statistical inference. The multilevel models we used included random effects at patient level to account for within-patient correlations, and robust clustered standard errors to account for within-clinic correlations due to patients nesting within same clinic. Letting Y_{it} denote the outcome measured at time t after the baseline for patient *I*, we estimated the following model:

$$(1) \quad Y_{it} = \beta_{0i} + \beta_1 Intervention_i + \beta_2 Time_t + \beta_3 Intervention_i * Time_t + \beta_4 (Time_t - t^*)$$
$$+ \beta_5 Intervention_i * (Time_t - t^*) + \beta_6 X_i + \varepsilon_{it}$$

where the patient-specific random intercept can be written as $\beta_{0i} = \alpha_{00} + \omega_{0i}$. We fitted linear models for continuous outcomes and logistic models for binary outcomes. Although scores on the ASI drug and ASI alcohol measures range from 0 to 1, participant scores on these measures were rescaled to a range of 0 to 100 prior to regression analyses (i.e., multiplied by 100). This adjustment was made to ensure meaningful regression estimates.

In the ITT analysis, individuals were assigned to the study arm to which they were randomized. Thus, $Intervention_i$ is equal to 1 if patient *i* is randomized to the intervention arm and 0 otherwise. $Time1_t$ is a continuous measure of time capturing differences in months between assessment *t* and assessment $t - 1$. Because the intervention ended by the time of the 6-month assessment, we centered time variables at 6-month follow-up in the following way: $Time_t$ equals to -4 for 2-month follow-up, -2 for 4-month follow-up, 0 for 6-*month* follow up, and 6 for 12-month follow-up. To model the pattern of outcome changes over time, our primary analysis employed linear spline models to divide the time axis into two segments, and within each segment consider piecewise linear trends (e.g., having a different time trend before and after the intervention ended). Specially, we denote t* to be the month when the intervention stopped, i.e., 6 months after baseline and $(Time_t - t^*)$ to be the post-intervention time trend, which equals to $(Time_t - t^*)$ if $Time_t > t^*$ and 0 otherwise. This choice of linear spline models allows for the ability to test whether the time trends differ before and after the intervention was finished and that treatment effect could attenuate over time once participants do not receive more intervention. Since the time variable is centered at 6-month follow-up, the beta coefficient on $Intervention_i$ can be interpreted as the treatment effect on outcome levels evaluated at the end of the intervention. That is, testing for the significance of $\beta_1$tests the hypothesis that the treatment was more effective than usual care in reducing substance use disorder and/or mental health illness, as evaluated at 6 months after the baseline. The coefficient of intervention by time interaction β_3 , tests whether the pattern of outcome over time would be no different between treatment and usual care groups. Similarly, β_5 tests whether changes in outcome responses over time was differential between treatment and control groups after the intervention had ended. The constant term α_{00} in model (1) represents the average level of outcome variables in the control group measured at the end of the intervention. The term ω_{0i} denotes the patient-specific random effect and ε_{ij} denotes the residual error term. X_i includes the baseline response of outcomes to control for severe differences at baseline and site indicators to adjust for variations by location. Because a successful randomized control trial balances both known and unknown confounders between treatment and control groups, our primary ITT analyses did not control for other covariates. We tested the robustness of the results by further adjusting for current medication use in a sensitivity analysis. The adjusted results did not differ from the main results (available upon request).

Our study sample is comprised of a broad clinical population, with a substantial number of patients who only had mild baseline symptoms in substance use and mental health. To test the hypothesis that intervention would be more effective among those with moderate to severe symptoms, we extended model (1) to include an intervention by baseline severity interaction. Baseline severity variable is dummy-coded, with 1 if participant's baseline outcome score equals or exceeds a moderate level of symptoms and 0 otherwise. Thresholds of moderate severity level are:

© 2019 Alegría et al. *JAMA Network Open.*

0.1 for ASI-Alcohol and ASI-Drug in their original scales, being positive to at least 1 substance on the urine drug test, 10 for PHQ-9, 10 for GAD-7, 33 for PCL-5, 1.5 for HSCL-20, 35 for the composite mental score and 20 for the composite substance score. These severity thresholds are based on either published literature or on the 50th percentile of the baseline outcome scores (except for the ASI-Drug measure). Because the distribution of ASI-Drug in this sample is highly rightly-skewed, 75th percentile of the baseline outcome scores was chosen to be the severity threshold. An interaction between this baseline severity dummy and intervention was then added into Model (1). With this adjustment, β_1 now represents the intervention effect at 6-month follow-up for those with mild symptoms. Coefficient of the baseline severity and intervention interaction measures the incremental effect of the treatment among participants with moderate to severe baseline symptoms. Results were discussed with further details in the manuscript.

We also examined whether effectiveness of the intervention was moderated by other person-level factors, such as income and recent immigration status, by estimating an interaction term between person-level factor and the intervention dummy. Income status was represented by a dummy coded variable for those whose self-reported personal income less than 15,000 US dollars, with medium to high income level as the reference category. Status of recent immigration was dummy-coded, with 1 if the length of residence in the host country was less than 5 years and 0 otherwise. Results show that effectiveness of the intervention was no different with respect to family income status. In contrast, we did observe a stronger treatment effect among recent immigrants in reducing mental health symptoms and substance use (results available upon request).

In the context of multisite randomized control trial, we tested whether the intervention was equally effective at three sites. To do this, we added an intervention by site interaction to Model (1) and tested if the interaction term was statistically significant, with Boston as the reference group (see eTable 3). We found that there was no evidence of differential effectiveness by site except for PHQ-9, where the intervention effect appears to be smaller in Madrid than Boston. Additionally, we tested whether the intervention effect was the same among patients who received the most sessions by telephone vs. in-person. We recategorized participants into four mutually exclusive groups: 1) patients in the control arm; 2) intervention patients who received zero sessions, 3) intervention patients who received most sessions by telephone, and 4) intervention patients who received most sessions in-person. When there was a tie between number of sessions received by telephone vs. in-person, we randomly assigned the patient into either group. Finally, we replaced the dummy-coded intervention variable with this multi-group variable to refit the ITT model (see eTable 4). For the study Latino immigrants with co-occurring problems, treatment in person appears more effective than by phone.

For the potential moderators (i.e., baseline severity level, site and type of treatment received), we also tested whether trajectories of treatment effects over time were moderated by the potential moderator. For instance, we estimated the difference in treatment time effects between those with moderate to severe symptoms at baseline vs those with mild symptoms using the interaction between the baseline severity indicator and treatment*time indicators. The statistically insignificant interaction coefficients indicated that the trajectory of treatment effects over time was not moderated by baseline severity level. Thus, primary moderation analysis did not include these additional interactions terms.

In secondary analyses, we used *dosage* (defined as number of treatment sessions received) as the independent variable of interest, categorized as dosage equal to zero (control group), 0-3 sessions (inadequate treatment for the intervention group), 4 or more sessions (adequate treatment for the intervention group). While this analysis no longer relies on random assignment, it serves to provide confirmation of the results from the intent-to-treat analysis and provides further estimates of the magnitude of the intervention effects on the main outcomes evaluated at 6 months after the baseline. Because the dosage analysis relies on actual treatment received, we used the original non-imputed sample for the analysis. To check the robustness of the *dosage* analysis, we conducted a third sensitivity analysis with an alternative categorization of treatment dosage. This alternative dosage variable has three categories: zero (control group), 0-5 sessions (intervention group) and 6 sessions or more (intervention group). We considered completion of treatment if the patient received 6 sessions or more, as 6 sessions cover the core components of the intervention (i.e. cognitive restructuring exercises, mindfulness practice, relapse prevention, etc.). Separating those with six or more sessions will allow us to examine the treatment effect for those who completed the core components of the intervention (see eTable 2).

© 2019 Alegría et al. *JAMA Network Open.*

We also performed separate sensitivity analyses to ensure the robustness of the results to alternative modeling strategies, estimation methods and how the missing data was handled.

Our first set of sensitivity analyses explored alternative methods for handling baseline response. Our primary analysis was carried out through analysis of covariance, which analyzes post-baseline responses, and makes an adjustment for the baseline response by including it as a covariate. In the sensitivity analysis, we retained the baseline response as part of the outcome vector and assumed the group means were equal at baseline, as is appropriate in a randomized control trial. The analytical data was extended to a longitudinal dataset, consisting of 5 repeated measurements per person, with baseline assessment included as an additional repeated assessment. To proceed, we re-estimated model (1) with this new dataset. Since baseline was added as additional time point, the $Time_t$ variable in this analysis, equals to -6 for baseline, -4, -2, 0 and 6 for the two, four, six and twelve-month assessments. Results of this sensitivity analysis are available upon request. We chose to present analysis of covariance as our primary results due to its potential efficiency gain. Because the baseline value has been obtained before any study intervention, i.e., the mean response that baseline is independent of treatment assignment, adjustment for baseline through covariance analysis will be more efficient, as it yields estimates of treatment effects with smaller standard errors[26].

Our primary ITT analysis modeled the nonlinear trend by fitting piecewise linear trends before and after treatment completion. We explored alternative modeling strategy in sensitivity analysis where the post-treatment trend in model (1) was replaced with a quadratic term of time trend to model the non-linear time trend and found similar results. Linear spline models were finally chosen because they generally provide a flexible way to accommodate many non-linear trends that cannot be approximated by simple polynomials in time[26].

Next, we checked the robustness of the results with respect to estimation method. Our primary ITT analysis used multilevel mixed-effect models to account for inter-participant correlation due to repeated measurement. Our sensitivity analysis instead used generalized estimating equations (GEE). GEE estimation will produce unbiased estimates if the underlying correlation structure is correctly specified. However, it has the limitation of being less efficient than a properly specified mixed model and does not allow weights to vary within clusters/panels[26].

We also performed a sensitivity analysis using a propensity score weighting approach to balance the treatment and control groups on residual differences within each site after randomization. To do so, we estimated a logistic regression model of membership in the treatment group conditional on baseline measures and interactions between baseline measures and site and generated the predicted probability (*phat*) of being assigned to the treatment group. In multivariate regression models, treatment group participants were given a weight of *1/phat* whereas control group participants were given a weight of *1*/1-*phat* to balance the groups on their predicted probability of being in the treatment group. Propensity score weights were then applied to all regression analysis. The weighted results remain similar to our main results.

Finally, we re-ran the *dosage* analysis with imputed data as a sensitivity check. In comparison to list-wise deletion, estimates of the imputed data from multiple imputation have slightly larger effect sizes (available from the authors upon request). This is because the intervention effects were greater among patients with moderate to severe symptomatology compared to those with mild symptomatology. Patients with missing observations, i.e., those who did not complete all follow-up assessments, had higher baseline symptom severity (eTable 5). Thus, by including these patients when using multiple imputation, the estimated intervention effect was amplified. Our primary analysis, which used non-imputed data and list-wise deletion, provides a conservative estimate of the actual effect of the treatment dosage.

© 2019 Alegría et al. *JAMA Network Open.*

eFigure 1. IIDEA study flow

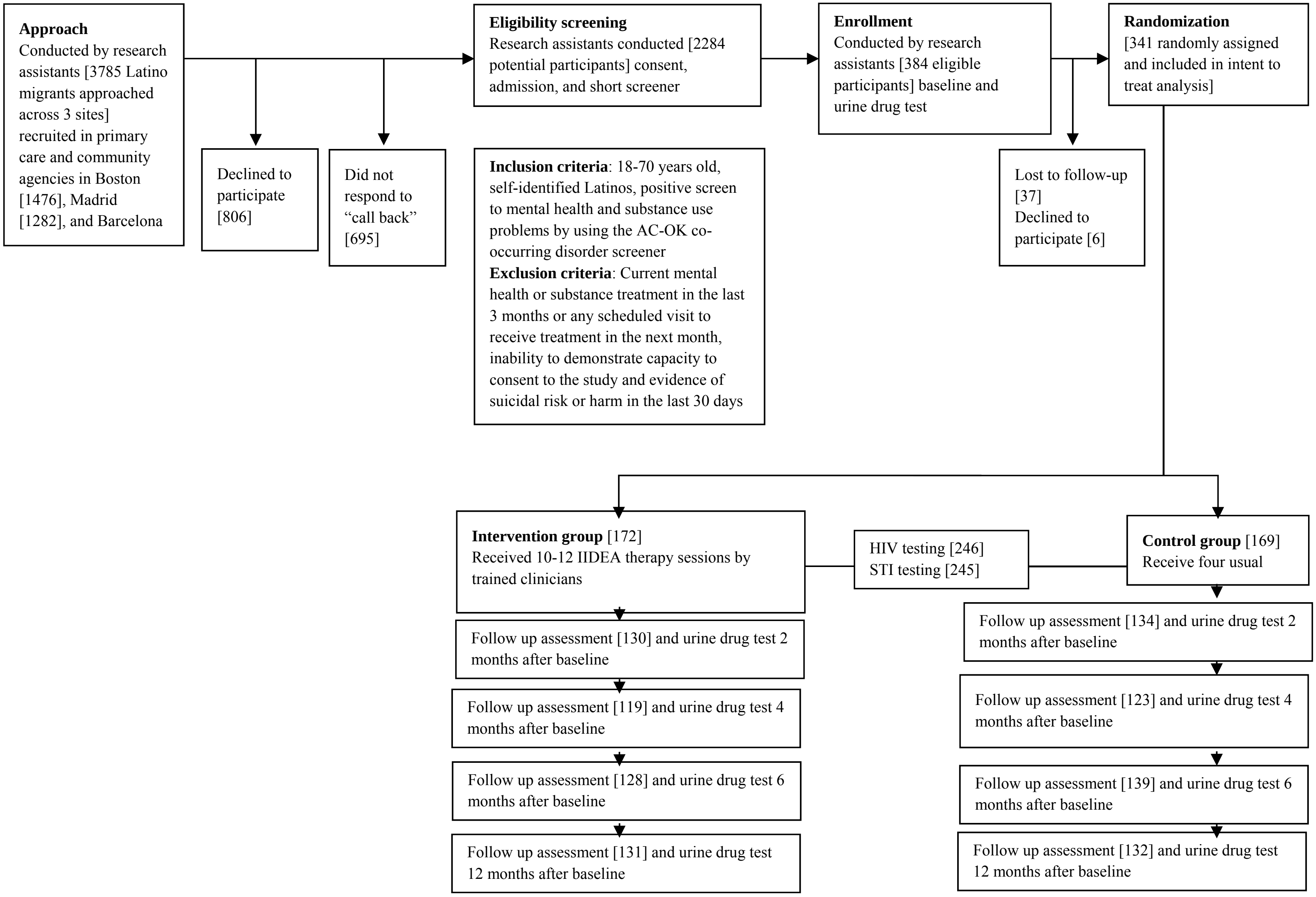

© 2019 Alegría et al. *JAMA Network Open.*

eFigure 2. Heterogenous treatment effects on primary and secondary outcomes across different subgroups

Panel A. Participants with Mild vs. Moderate to Severe Symptoms at Baseline

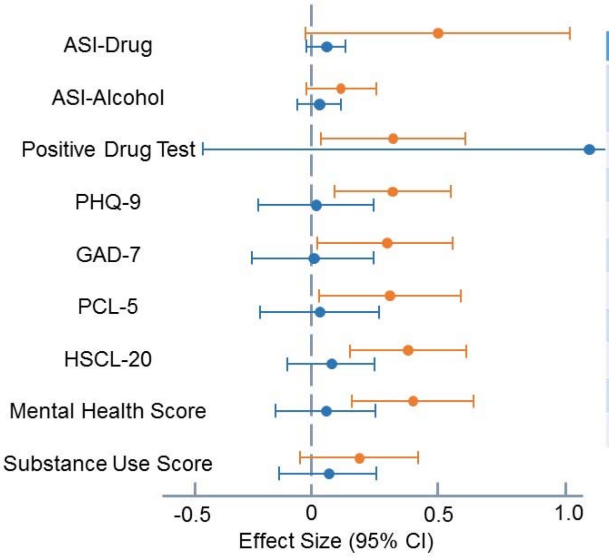

Effect Size (95% CI)		
Outcomes	Mild Symptoms	Moderate to Severe Symptoms
ASI-Drug	0.07 (-0.05-0.18)	0.52 (-0.06-1.10)
ASI-Alcohol	0.01 (-0.14-0.16)	0.10 (-0.05-0.26)
Drug Test	1.29 (0.53-3.15)	0.25 (0.09-0.67)
PHQ-9	0.02(-0.22-0.25)	0.38 (0.15-0.62)
GAD-7	0.00 (-0.29-0.29)	0.34 (0.06-0.63)
PCL-5	0.05(-0.23-0.34)	0.37 (0.08-0.65)
HSCL-20	0.06 (-0.17-0.29)	0.44 (0.21-0.67)
MH Score	0.05 (-0.22-0.31)	0.44 (0.18-0.71)
SU Score	0.06 (-0.20-0.31)	0.16 (-0.10-0.41)

● Mild Symptoms ● Moderate to Severe Symptoms

Panel B. Recent Migrants vs. Non-Recent Migrants

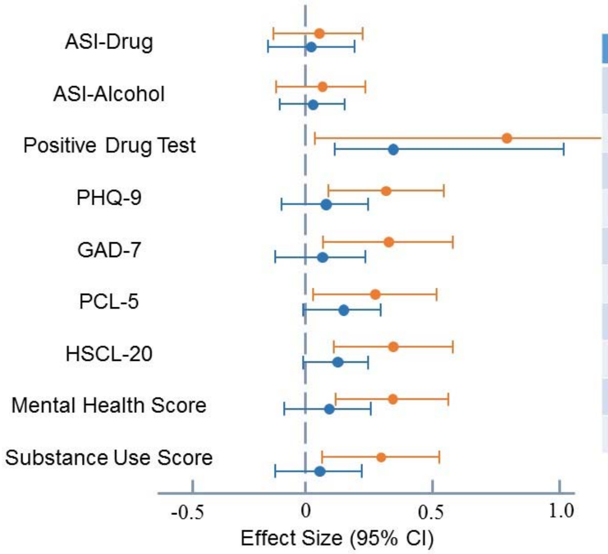

Effect Size (95% CI)		
Outcomes	Non-Recent Migrants	Recent Migrants
ASI-Drug	0.02 (-0.17-0.21)	0.08 (-0.11-0.27)
ASI-Alcohol	0.04 (-0.09-0.16)	0.11(-0.08-0.31)
Drug Test	0.85 (0.3-2.05)	0.34 (0.11-1.07)
PHQ-9	0.13 (-0.04-0.31)	0.38 (0.13-0.62)
GAD-7	0.04 (-0.16-0.25)	0.36 (0.09-0.64)
PCL-5	0.14 (-0.06-0.35)	0.30 (0.04-0.56)
HSCL-20	0.19 (-0.01-0.28)	0.41 (0.17-0.66)
MH Score	0.11 (-0.09-0.31)	0.40 (0.14-0.65)
SU Score	0.03 (-0.16-0.21)	0.28 (0.04-0.52)

● Non-Recent Migrants ● Recent Migrants

Note: Effect sizes are calculated in the metric of Odds Ratios for binary outcomes and in the metric of Cohen's d for continuous outcomes. Positive drug test is a binary outcome based on the results of urine drug tests for use of any six drug types (amphetamines, benzodiazepines, cocaine, methamphetamine, heroin and marijuana). ASI: Addition Severity Index; PHQ: Patient Health Questionnaire; GAD: Generalized Anxiety Disorder; PCL: Post-Traumatic Stress Disorder Checklist; HSCL: Hopkins Symptom Checklist; DAST: Drug Abuse Screening Test; AUDIT: Alcohol Use Disorders Identification Test; BDEPQ: Benzodiazepine Dependence Questionnaire.

© 2019 Alegría et al. *JAMA Network Open.*

eTable 1. List of Assessment Measures/Variables

**Socio-Demographic variables**	
Demographics	Site (Boston/Madrid/Barcelona), age (18-34, 35-49, 50+), gender (male/female), race/ethnicity (White, Black, Indigenous/Native American, Hispanic/Latino/Caribbean, Mixed), education level (less than high school/HS Diploma, GED, vocational school, or more), Total Personal Income Last Year (< 15000 US Dollars or ≥ 15000 US Dollars)
**Clinical Measures**	
Depression - Patient Health Questionnaire (PHQ-9)	9-item screening questionnaire to determine severity of depressive symptoms. Internal consistency was α= 0.85. Spanish version has good agreement with independent mental health professional Dx (k = 0.74; overall accuracy, 88%; sensitivity, 87%; specificity, 88%) (27).
Generalized Anxiety - General Anxiety Disorder 7-item screener (GAD-7)	7-item screening tool and severity measure for generalized anxiety disorder. In Spanish, a cut-off point of 10 showed adequate values for sensitivity (86.8%) and specificity (93.4%); AUC statistically significant [AUC = 0.957-0.985; p < 0.001]; Internal consistency was α= 0.86 (28)
Post-traumatic Stress Disorder - PTSD Checklist for DSM-5 (PCL-5)	The PCL-5 is a 20-item questionnaire, corresponding to the *DSM-5* symptom criteria for PTSD. We consider 33 as a cutoff given the latest data. The internal consistency was α= 0.94 (29)
Drug Abuse - Drug Abuse Screening Test (DAST-10)	10-item self-report instrument that has been condensed from the 28-item DAST. It is designed for clinical screening of substance use. The DAST-10 yields a quantitative index of the degree of consequences related to drug abuse. (α= 0.87) (30)
Alcohol Abuse – Alcohol Use Disorders Identification Test – C (AUDIT-C)	World Health Organization screener for excessive drinking. 3-question screen that evaluates lifetime and past 30-day alcohol use behaviors (α=0.78) (31).
Benzodiazepines - Benzodiazepine Dependence Questionnaire (BDEPQ)	The BDEPQ is designed to measure dependence on benzodiazepine tranquilizers, sedatives, and hypnotics. We selected 10 representative items from this questionnaire. (α = 0.89) (32)
Alcohol – Addiction Severity Index Lite (ASI Lite) – Alcohol and Drug - Addiction Severity Index Lite (ASI Lite) - Drug	Shortened version of the Addiction Severity Index (ASI) which obtains lifetime information about problem behaviors, as well as problems within the previous 30 days. We used drug and alcohol questions only (α=0.84 for alcohol and α=0.70 for drugs) (33).
HSCL-20 – Hopkins Symptom Checklist-20	The HSCL-20 is a symptom inventory for depression that indicates overall mental health symptoms. Internal consistency was α=0.94 (34).
Mindfulness Awareness - Mindfulness Awareness Attention Scale	The MAAS is a 15-item scale designed to assess a core characteristic of dispositional mindfulness, namely, open or receptive awareness of and attention to what is taking place in the present. The scale shows strong

© 2019 Alegría et al. *JAMA Network Open.*

	psychometric properties and has been validated with community samples. (α = 0.87) (35, 36).
Smoking - Fagerstrom Test for Nicotine Dependence	The Fagerstrom Test is a standard instrument for assessing the intensity of physical addiction to nicotine, and designed to provide an ordinal measure of nicotine dependence related to cigarette smoking. It contains six items that evaluate the quantity of cigarette consumption, the compulsion to use, and dependence (α =0.75) (37).
Trauma Exposure - Brief Trauma Questionnaire (BTQ)	10-item self-report measure that examines experiences with potentially traumatic events that would meet Criterion A (serious injury/life threat/subjective response) for PTSD diagnosis according to DSM-IV. It is derived from the Brief Trauma Interview. Internal consistency was (38).
Any positive in Urine Test	Using the DrugCheck for drug metabolites with a binary outcome (yes/no) for use of any of this six drug types: amphetamines, benzodiazepines, cocaine, methamphetamine, marijuana, and heroin
**Cultural/Social Factors**	
Citizenship (Yes/No)	Immigration status was defined by whether the participant was a citizen or non-citizen.
Years in US/Spain	
Number of Home Visits in the Past 12 Months	
Discrimination Scale	Everyday Discrimination Scale (39) (α=0.82). The scale measures chronic and routine unfair treatment in everyday life. Adopted from the Detroit Area Study and National Latino and Asian-American Study (NLAAS). Participants were asked 9 questions pertaining to discrimination that affect Latinos and other minorities.
Ethnic Identity Scale	The 3-item Ethnic Identity Scale (α = 0.73), derived from the 35-item Cultural Identity Scale for Latino Adolescents (40), was used to gauge cultural identity. Questions ask about what culture or ethnic/racial groups an individual identifies with.
Intercultural/Family Conflict Scale	We used 3 items of the Family/Culture Stress subscale of the Hispanic Stress Inventory (HIS) (α=0.66), designed to measure family interference with personal goals, arguments with family members, and the breakdown of the family unit (41-43).
Sense of Belonging	One question derived from qualitative research from the research team in previous studies (44). The question is: You feel like you don't belong either in your Latino country or in the U.S.?

© 2019 Alegría et al. *JAMA Network Open.*

Recruitment Site	
Recruitment Site	Primary Care Clinics/Community Sites/Emergency Room/Patient Referrals

CHANGES FROM THE ORIGINAL PROTOCOL:

We updated measures from the originally registered protocol: We replaced the Posttraumatic Cognitions Inventory with the PTSD Checklist for DSM-5 to follow the most updated DSM-V guidelines. We also added the Hopkins Symptoms Checklist after the registration of the trial to provide information about overall mental health symptoms/psychological distress. We replaced our originally registered follow-up assessment schedule (1, 3, 6 months) with 2, 4, 6, and 12-month assessments, to better align with intervention flow, at the recommendation of our Advisory Board.

© 2019 Alegría et al. *JAMA Network Open.*

eTable 2. Analysis of participation rates (receiving 6+ sessions) in IIDEA intervention on main outcomes at 6-month follow-up (N=341)[a]

	ASI-Drug	ASI-Alcohol	Positive in Urine Drug Test	PHQ-9	GAD-7	PCL-5	HSCL-20	Composite Mental Score	Composite Substance Score
Intervention Patients with 0-5 Sessions	1.09	0.97	0.01	-0.92	0.33	-1.90	-0.07	-1.16	2.43
	(0.87)	(1.44)	(0.47)	(0.55)	(0.67)	(1.45)	(0.06)	(2.05)	(1.73)
Intervention Patients with 6 + Sessions	-1.50*	0.61	-0.83	-1.70*	-1.36*	-4.74*	-0.30**	-6.40*	-4.30**
	(0.67)	(1.60)	(0.62)	(0.74)	(0.58)	(2.14)	(0.11)	(2.42)	(1.25)
Madrid[b]	-1.11	-2.22	-0.35	-0.18	-0.36	-2.15	-0.01	-1.64	1.78
	(1.15)	(2.56)	(0.59)	(0.73)	(0.72)	(1.73)	(0.14)	(2.39)	(1.75)
Barcelona	-1.18	-0.61	-0.52	-1.46*	-1.39**	-5.48***	-0.18	-5.76**	0.64
	(0.83)	(1.55)	(0.36)	(0.66)	(0.41)	(1.22)	(0.10)	(1.68)	(1.09)
Baseline Outcome	0.62***	0.42***	2.25***	0.47***	0.45***	0.51***	0.48***	0.53***	0.55***
	(0.08)	(0.05)	(0.36)	(0.05)	(0.06)	(0.05)	(0.06)	(0.06)	(0.06)
Constant	1.61	4.79*	-1.49***	3.64***	2.69***	9.25***	0.42**	10.20**	3.64*
	(0.94)	(2.07)	(0.32)	(0.84)	(0.73)	(1.69)	(0.14)	(2.92)	(1.77)
N	268	268	257	268	268	268	216	268	268

[a] This analysis only uses data from the 6-month follow up assessment. Missing data is list-wise deleted. The analytical sample for each regression slightly varies as the missing pattern of each outcome variable slightly differs.

[b] The reference group is patients in control arm and the reference site is Boston.

Standard errors are reported in parentheses. Coefficients in terms of the log odds are reported for binary outcome.

*= p<0.05; **=p<0.01; ***=p<0.001.

ASI: Addition Severity Index; PHQ: Patient Health Questionnaire; GAD: Generalized Anxiety Disorder; PCL: Post-Traumatic Stress Disorder Checklist; HSCL: Hopkins Symptom Checklist; DAST: Drug Abuse Screening Test; AUDIT: Alcohol Use Disorders Identification Test; BDEPQ: Benzodiazepine Dependence Questionnaire.

© 2019 Alegría et al. *JAMA Network Open.*

eTable 3. Intent-to-treat analysis of IIDEA intervention on main outcomes *by site* (N=341) [a]

	ASI-Drug	ASI-Alcohol	Positive in Urine Drug Test	PHQ-9	GAD-7	PCL-5	HSCL-20	Composite Mental Health	Composite Substance Use
Intervention	-0.83	0.15	-0.70	-2.04*	-1.85	-6.00*	-0.32*	-7.93*	-2.37
	(1.71)	(1.62)	(0.63)	(0.90)	(1.00)	(2.46)	(0.14)	(3.25)	(2.62)
Time[b]	-0.13	-0.97**	0.01	-0.38***	-0.43***	-1.36***	-0.06***	-1.72***	-0.52*
	(0.16)	(0.34)	(0.10)	(0.11)	(0.10)	(0.32)	(0.02)	(0.37)	(0.26)
Intervention*Time	0.11	0.46	-0.05	-0.05	0.10	0.44	-0.01	0.28	0.01
	(0.17)	(0.39)	(0.14)	(0.15)	(0.13)	(0.50)	(0.02)	(0.53)	(0.40)
(Time-t*)[c]	0.10	0.96*	-0.04	0.25	0.42**	1.02*	0.06**	1.40**	0.54
	(0.21)	(0.42)	(0.14)	(0.16)	(0.14)	(0.41)	(0.02)	(0.51)	(0.34)
Intervention*(Time-t*)	-0.12	-0.53	0.10	0.21	-0.08	-0.11	0.02	0.09	-0.17
	(0.26)	(0.52)	(0.22)	(0.20)	(0.19)	(0.64)	(0.03)	(0.71)	(0.53)
Madrid[d]	-0.90	0.49	-1.22*	-0.95	-1.21	-5.21**	-0.17	-5.09*	1.38
	(1.33)	(1.87)	(0.51)	(0.62)	(0.74)	(1.75)	(0.11)	(2.27)	(2.09)
Barcelona	-0.76	-0.20	-0.34	-0.94	-1.62**	-5.45**	-0.20*	-5.35**	1.44
	(1.22)	(1.23)	(0.52)	(0.56)	(0.61)	(1.71)	(0.10)	(1.97)	(1.77)
Intervention*Madrid[e]	1.14	-0.08	1.32	1.89*	1.85	5.07	0.24	7.34*	2.15
	(1.72)	(2.49)	(0.78)	(0.95)	(1.03)	(2.95)	(0.14)	(3.53)	(2.79)
Intervention*Barcelona	1.05	-0.28	0.08	0.76	1.31	2.52	0.12	4.10	1.09
	(1.63)	(1.69)	(0.67)	(0.87)	(0.91)	(2.41)	(0.13)	(3.02)	(2.68)
Baseline Outcome	0.56***	0.47***	3.55***	0.53***	0.49***	0.55***	0.45***	0.58***	0.51***
	(0.04)	(0.04)	(0.42)	(0.04)	(0.04)	(0.04)	(0.05)	(0.04)	(0.04)
Constant	2.39	6.83***	-2.24***	3.19***	3.31***	10.49***	0.58***	10.89***	6.18**
	(1.27)	(1.48)	(0.50)	(0.67)	(0.74)	(1.95)	(0.11)	(2.62)	(2.00)

[a] This analysis uses longitudinal data of 341 participants with four follow-up assessments per participant. Each outcome variable was measured four times at 2 months, 4 months, 6 months and 12 months follow-up, respectively. The unit observation is a specific follow-up assessment. Missing data is handled by multiple imputation method (eAppendix 3, supplement).
[b] *Time* is a continuous variable which equals to -4, -2, 0 and 6 for RA2 to RA5, respectively.
[c] *Time-t** is a continuous variable equal to 6 for RA5 and 0 otherwise. *t** denotes the time when treatment ends which equals to 6.
[d] The reference group is patients in control arm and the reference site is Boston.
[e] *Intervention*Madrid* represents the differences in intervention effect among Madrid patients as compared to Boston.
Standard errors are reported in parentheses. Coefficients in terms of the log odds are reported for binary outcome.
*= p<0.05; **=p<0.01; ***=p<0.001.
ASI: Addition Severity Index; PHQ: Patient Health Questionnaire; GAD: Generalized Anxiety Disorder; PCL: Post-Traumatic Stress Disorder Checklist; HSCL: Hopkins Symptom Checklist; DAST: Drug Abuse Screening Test; AUDIT: Alcohol Use Disorders Identification Test; BDEPQ: Benzodiazepine Dependence Questionnaire.

© 2019 Alegría et al. *JAMA Network Open.*

eTable 4. Intent-to-treat analysis of IIDEA intervention on main outcomes *by received intervention type* (N=341)[a]

	ASI-Drug	ASI-Alcohol	Positive in Urine Drug Test	PHQ-9	GAD-7	PCL-5	HSCL-20	Composite Mental Score	Composite Substance Score
Intervention Patients with 0 sessions (ref=Control Patients)[b]	1.80**	2.31*	0.46	0.11	0.71	0.86	0.03	1.88	3.87***
	(0.56)	(1.13)	(0.50)	(0.44)	(0.46)	(1.20)	(0.06)	(1.49)	(1.04)
Intervention Patients with Most Sessions Received by Telephone	0.08	-1.65	0.02	-0.33	-0.43	-4.71*	-0.10	-3.35+	-1.78
	(0.71)	(1.84)	(0.53)	(0.57)	(0.52)	(1.87)	(0.10)	(1.96)	(1.68)
Intervention Patients with Most Sessions Received in Person	-1.07*	-1.85*	-0.63*	-1.45***	-1.61***	-5.06***	-0.29***	-6.41***	-3.62***
	(0.50)	(0.94)	(0.28)	(0.42)	(0.44)	(1.33)	(0.07)	(1.59)	(1.09)
Time	-0.05	-0.75**	-0.01	-0.42***	-0.39***	-1.17***	-0.07***	-1.62***	-0.50**
	(0.11)	(0.27)	(0.06)	(0.07)	(0.07)	(0.20)	(0.01)	(0.24)	(0.18)
(Time-t*)	0.01	0.72*	-0.00	0.39***	0.39***	1.02***	0.07***	1.53***	0.46*
	(0.13)	(0.33)	(0.09)	(0.11)	(0.09)	(0.28)	(0.01)	(0.34)	(0.23)
Madrid[b]	-1.11	-2.22	-0.35	-0.18	-0.36	-2.15	-0.01	-1.64	1.78
	(1.15)	(2.56)	(0.59)	(0.73)	(0.72)	(1.73)	(0.14)	(2.39)	(1.75)
Barcelona	-1.18	-0.61	-0.52	-1.46*	-1.39**	-5.48***	-0.18	-5.76**	0.64
	(0.83)	(1.55)	(0.36)	(0.66)	(0.41)	(1.22)	(0.10)	(1.68)	(1.09)
Baseline Outcome	0.62***	0.42***	2.25***	0.47***	0.45***	0.51***	0.48***	0.53***	0.55***
	(0.08)	(0.05)	(0.36)	(0.05)	(0.06)	(0.05)	(0.06)	(0.06)	(0.06)
Constant	1.61	4.79*	-1.49***	3.64***	2.69***	9.25***	0.42**	10.20**	3.64*
	(0.94)	(2.07)	(0.32)	(0.84)	(0.73)	(1.69)	(0.14)	(2.92)	(1.77)

[a] This analysis uses longitudinal data of 341 participants with four follow-up assessments per participant. Each outcome variable was measured four times at 2 months, 4 months, 6 months and 12 months follow-up, respectively. The unit observation is a specific follow-up assessment. Missing data is handled by multiple imputation method (eAppendix 3, supplement).

[b] The reference group is patients in control arm and the reference site is Boston.

Standard errors are reported in parentheses. Coefficients in terms of the log odds are reported for binary outcome.

*= p<0.05; **=p<0.01; ***=p<0.001.

ASI: Addition Severity Index; PHQ: Patient Health Questionnaire; GAD: Generalized Anxiety Disorder; PCL: Post-Traumatic Stress Disorder Checklist; HSCL: Hopkins Symptom Checklist; DAST: Drug Abuse Screening Test; AUDIT: Alcohol Use Disorders Identification Test; BDEPQ: Benzodiazepine Dependence Questionnaire.

© 2019 Alegría et al. *JAMA Network Open.*

eTable 5. Statistically Significant baseline differences[a] between participants with missing/incomplete assessments and participants with complete assessments (N=341)

	Participants with Complete Assessments (N=197)	Participants with Incomplete Assessments (N=144)	χ^2 or t Test	P Value
DAST, Mean (SD)	1.15 (2.11)	1.65 (2.52)	-1.99	0.048
Education Level, No. (%)				
Less than High School	60 (30.5)	71 (49.3)	12.49	0.001
High School Diploma, General Education Diploma, Vocational School, or More	137 (69.5)	73 (50.7)		
Recruitment Site, No. (%)				
Primary Care Clinics	77 (39.1)	71 (49.3)	8.55	0.036
Community Sites	56 (28.4)	22 (15.3)		
Emergency Room	11 (5.6)	8 (5.6)		
Patient Referrals	53 (26.9)	43 (29.9)		
Number of Children, No. (%)				
None	138 (70.1)	85 (59.0)	4.47	0.035
One and More	59 (29.9)	59 (41.0)		

[a] All baseline characteristics reported in the manuscript Table 1 are compared across the two groups. Only ones with statistically significant differences are reported.

© 2019 Alegría et al. *JAMA Network Open.*

References

1. Chavez LM, Shrout PE, Wang Y, Collazos F, Carmona R, Alegría M. Evaluation of the AC-OK mental health and substance abuse screening measure in an international sample of Latino immigrants. *Drug Alcohol Depend.* 2017;180:121-128.
2. Gibbons R, Alegría M, Cai L, et al. Successful validation of the CAT-MH Scales in a sample of Latin American migrants in the United States and Spain. *Psychol Assess.* 2018.
3. Gibbons RD, Weiss DJ, Frank E, Kupfer D. Computerized adaptive diagnosis and testing of mental health disorders. *Annu Rev Clin Psychol.* 2016;12:83-104.
4. Alegría M, Canino G, Ríos R, et al. Inequalities in use of specialty mental health services among Latinos, African Americans, and non-Latino whites. *Psychiatr Serv.* 2002;53(12):1547-1555.
5. Alegría M, Chatterji P, Wells K, et al. Disparity in Depression Treatment among Racial and Ethnic Minority Populations in the United States. *Psychiatr Serv.* 2008;59(11):1264-1272.
6. Alegría M, Mulvaney-Day N, Torres M, Polo A, Cao Z, Canino G. Prevalence of psychiatric disorders across Latino subgroups in the United States. *Am J Public Health.* 2007;97(1):68-75.
7. Alegría M, Takeuchi D, Canino G, et al. Considering context, place and culture: the National Latino and Asian American Study. *Int J Methods Psychiatr Res.* 2004;13(4):208-220.
8. Wells K, Klap R, Koike A, Sherbourne C. Ethnic disparities in unmet need for alcoholism, drug abuse, and mental health care. *Am J Psychiatry.* 2001;158(12):2027-2032.
9. Boyd JE, Lanius RA, McKinnon MC. Mindfulness-based treatments for posttraumatic stress disorder: a review of the treatment literature and neurobiological evidence. *Journal of psychiatry & neuroscience : JPN.* 2017;42(6):170021.
10. Fortuna LR, Porche MV, Padilla A. A treatment development study of a cognitive and mindfulness-based therapy for adolescents with co-occurring post-traumatic stress and substance use disorder. *Psychology and psychotherapy.* 2017.
11. Hofmann SG, Gomez AF. Mindfulness-Based Interventions for Anxiety and Depression. *The Psychiatric clinics of North America.* 2017;40(4):739-749.
12. Teasdale JD, Segal ZV, Williams JMG, Ridgeway VA, Soulsby JM, Lau MA. Prevention of relapse/recurrence in major depression by mindfulness-based cognitive therapy. *J Consult Clin Psychol.* 2000;68(4):615.
13. Fortuna LR, Porche MV, Padilla A. A treatment development study of a cognitive and mindfulness-based therapy for adolescents with co-occurring post-traumatic stress and substance use disorder. *Psychology and psychotherapy.* 2017.
14. Mueser KT, Rosenberg SD, Xie H, et al. A randomized controlled trial of cognitive-behavioral treatment for posttraumatic stress disorder in severe mental illness. *J Consult Clin Psychol.* 2008;76(2):259-271.
15. Mueser KT, Rosenberg SD, Goodman LA, Trumbetta SL. Trauma, PTSD, and the course of severe mental illness: an interactive model. *Schizophr Res.* 2002;53(1-2):123-143.
16. El-Bassel N, Witte SS, Gilbert L, et al. The efficacy of a relationship-based HIV/STD prevention program for heterosexual couples. *Am J Public Health.* 2003;93(6):963-969.
17. Pettifor A, MacPhail C, Nguyen N, Rosenberg M, Parker L, Sibeko J. Feasibility and acceptability of Project Connect: a couples-based HIV-risk reduction intervention among young couples in Johannesburg, South Africa. *AIDS care.* 2014;26(4):476-482.
18. Wu E, El-Bassel N, McVinney LD, et al. Feasibility and promise of a couple-based HIV/STI preventive intervention for methamphetamine-using, black men who have sex with men. *AIDS Behav.* 2011;15(8):1745.
19. Bolu OO, Lindsey C, Kamb ML, et al. Is HIV/sexually transmitted disease prevention counseling effective among vulnerable populations?: a subset analysis of data collected for a randomized, controlled trial evaluating counseling efficacy (Project RESPECT). *Sex Transm Dis.* 2004;31(8):469-474.
20. Kamb ML, Dillon BA, Fishbein M, Willis KL. Quality assurance of HIV prevention counseling in a multi-center randomized controlled trial. Project RESPECT Study Group. *Public Health Rep.* 1996;111(Suppl 1):99.
21. Metcalf CA, Douglas Jr JM, Malotte CK, et al. Relative efficacy of prevention counseling with rapid and standard HIV testing: a randomized, controlled trial (RESPECT-2). *Sex Transm Dis.* 2005;32(2):130-138.
22. Horsfall J, Cleary M, Hunt GE, Walter G. Psychosocial treatments for people with co-occurring severe mental illnesses and substance use disorders (dual diagnosis): A review of empirical evidence. *Harv Rev Psychiatry.* 2009;17(1):24-34.

© 2019 Alegría et al. *JAMA Network Open.*

23. *Stata Statistical Software: Release 14* [computer program]. College Station, TX: StataCorp LP; 2015.
24. Rubin D. *Multiple Imputation for Nonresponse in Surveys* New York: John Wiley & Sons 1987.
25. Raghunathan TE, Lepkowski JM, Van Hoewyk J, Solenberger P. A multivariate technique for multiply imputing missing values using a sequence of regression models. *Surv Methodol.* 2001;27(1):85-96.
26. Fitzmaurice GM, Laird NM, Ware JH. Modeling the Mean: Analyzing Response Files. In: *Applied longitudinal analysis.* Vol 998. Hoboken, NJ: John Wiley & Sons; 2012.

© 2019 Alegría et al. *JAMA Network Open.*
