# Supplementary material for: Evaluation of the Integrated Intervention for Dual Problems and Early Action Among Latino Immigrants With Co-occurring Mental Health and Substance Misuse Symptoms: A Randomized Clinical Trial
Source: JAMA Netw Open. 2019 Jan 11;2(1):e186927. doi: 10.1001/jamanetworkopen.2018.6927 (PMC6484537; doi:10.1001/jamanetworkopen.2018.6927)
Supplement: Supplement 2. — eAppendix 1. Study Design of the IIDEA RCT eAppendix 2. Description of the Intervention and Fidelity eAppendix 3. Analytical Methods—Data Preparation eAppendix 4. Analytical Methods—Data Analysis eFigure 1. IIDEA Study Flow eFigure 2. Heterogenous Treatment Effects on Substance Misuse Outcomes Across Different Subgroups eTable 1. List of Assessment Measures/Variables eTable 2. Analysis of Participation Rates (Receiving 6+ Sessions) in IIDEA Intervention on Main Outcomes at 6-Month Follow-up eTable 3. Intent-to-Treat Analysis of IIDEA Intervention on Main Outcomes by Site eTable 4. Intent-to-Treat Analysis of IIDEA Intervention on Main Outcomes by Received Intervention Type eTable 5. Significant Baseline Differences Between Participants With Missing/Incomplete Assessments and Participants With Complete Assessments [file jamanetwopen-2-e186927-s002.pdf]

International Latino Research Partnership (ILRP)

STUDY PROTOCOL (Based on the Original Study Protocol)

April 2018

PI: Margarita Alegria, Ph.D.
Funder: National Institute on Drug Abuse (NIDA) of the National Institutes of Health,
Award Number R01DA034952

TABLE OF CONTENTS:

A. Background and Significance …………………………………………………. 3

B. Study Design Summary …………………………………………………….. 4

C. Study Population …………………………………………………………… 5

D. Study Setting ………………………………………………………………... 5

E. Sampling, Recruitment and Consent ……………………………………….... 6

F. Research Procedures ……………………………………………………… 7

G. Intervention ……………………………………………………………………. 11

H. Analytical Methods ……………………………………………………………. 15

I. Safety …………………………………………………………………………...22

J. Monitoring, Quality assurance and Ethics ……………………………………….. 23

References ………………………………………………………………………… 25

A. BACKGROUND AND SIGNIFICANCE

The International Latino Research Partnership (ILRP) study aimed to provide evidence to increase accessibility, quality and outcomes of integrated behavioral health care for migrant Latinos with co-occurring substance misuse and mental health problems. The ILRP focused on closing the treatment gap now prominent on the international health agenda, with calls for global initiatives to improve access to behavioral health treatment worldwide.[1,2] Untreated substance use disorders are associated with premature mortality,[3,4] productivity loss,[5] high rates of disability,[6,7] and increased risk for HIV.[8-12] Drug use that does not initially comply with criteria for abuse and dependence places users at risk for co-occurring mental health problems such as elevated depression symptoms,[13-16] high levels of anxiety [6,17-19] and HIV risk. In reciprocal fashion, having mental health problems radically increases the likelihood of augmented drug use[20-22] and problematic alcohol use (i.e., binge drinking and heavy episodic drinking),[23-25] and is highly comorbid with smoking.[26-28] Yet, a review of 59 dual disorder treatment studies concludes that “the current status of the literature, unfortunately, is so poor that urgent attention by funding agencies is needed to conduct more methodologically rigorous research in this area, given the high prevalence of dual-diagnosis patients. Funding agencies should make studies in this area a high priority and focus on funding high-quality studies (pg. 534)”.[29] Globally, most people with substance use and/or mental health problems receive no treatment.[30-35] This gap requires "a comprehensive response”[36] through an International Research Collaboration in Drug Addiction Research.

Compounding international challenges in delivering quality behavioral health services[37,38] are worldwide population movements for economic opportunity,[39,40] one element of which brings unprecedented numbers of Latino migrants (defined as immigrants as well as transnational migrants who move between the country of origin and host country)[41] from Latin America to host countries like Spain and the U.S.[42,43] Three primary scientific reasons led us to select Spain as our collaborating site. First, Spain has the largest number of Latino migrants in the European Union,[44] where most immigrants are Latino and, in numbers, it is only surpassed by the U.S..[45,46] Migrants comprise 12.2% of Spain’s population, with close to a third (28%) being Latinos from the Caribbean, Central and South America.[44] Migrants in the U.S. represent 13% of the U.S. population and 53% of these are Latino.[45] As Latino migrants attempt to acculturate to the host

country, they drink more alcohol[47] and use more drugs,[47-50] straining the health care system, public safety, and increasing their risk for HIV.[51,52] In Barcelona, Latino migrants are the second largest population seeking services for drug dependency apart from native Spaniards.[53] Yet, this accelerated growth of Latino migrants has not been paralleled with the development of knowledge of how to better serve immigrant/migrant Latino populations. Migrant Latinos confront elevated barriers to behavioral health care,[48,54] and require service transformations to ameliorate unmet need.

The ILRP project provided the opportunity to investigate and describe the differential service needs of Latino migrants, as well as factors that might confer differential risk for co-occurring behavioral health problem. A key element of this work was adapting and testing the feasibility, acceptability and efficacy of the "Integrated Intervention for Dual Problems and Early Action" (IIDEA) intervention. The IIDEA intervention addresses mental health, substance misuse, and prevention of HIV/STIs, for Latino participants identified in primary care, community sites and emergency departments across three sites. This clinical trial was conducted in Massachusetts as well as at two sites in Spain. As such, the proposed ILRP multi-site international project is a critical step towards developing models of integrated care for the large and diverse Latino migrant population and more broadly towards understanding how best to integrate evidence-based assessment and treatments for co-occurring substance and mental health problems and HIV/STI risks.

B. STUDY DESIGN SUMMARY

We conducted the IIDEA randomized clinical trial between September 2014 and February 2017, with Latino migrants in Massachusetts and at two sites in Spain. Based on the promising results of the AC-OK as a screener for dual diagnosis,[55] we used the AC-OK as the screening tool for the clinical trial. Eligible participants needed to endorse two mental health items and two substance use items on the AC-OK, and not be in specialty care in the past 3 months or have an appointment planned. The trial design included five research assessments: baseline, and follow up assessment at two months, four-months, six-months, and twelve-months from baseline. Participants were also administered a Computerized Adaptive Testing interview for mental health (CAT-MH), funded by a research supplement (NIMH; PI: Gibbons). Participants were

randomization into either a 10-session intervention or a control group that received regular check-ins with a care manager. Participants were also provided access to HIV and STI testing by the study team and urine testing for drug metabolites as part of administrative supplement to the NIDA grant (PI: Alegria).

C. STUDY POPULATION

Participants: Across all sites, 360 patients were targeted to be randomized into the control and intervention arms of the study, to achieve a target sample size of at least 300 assuming attrition of 20%. We successfully finalized the study sample with 341 participants, having screened 2284 participants across study sites to arrive at this number.

C.1 Eligibility criteria: All participants were self-reported 1st or second generation Latinos that spoke English or Spanish, and were between the ages of 18-70. To be eligible for the intervention, participants had to screen positive to both substance use and mental health problems in the AC-OK (AC– Andrew Cherry and OK – Oklahoma) screener[56]. Participants needed to have 2 positive mental health scores and 2 positive substance misuse scores. Patients could enroll in the study if they were taking psychotropic medications prescribed by their primary care clinician but not seeing a psychiatrist or psychologist for therapy.

C.2 Exclusion criteria: Participants were excluded if there was evidence of any of the following: (1) currently receiving behavioral health services (i.e. have an appointment scheduled in coming month) or recent (last 3 months) substance use treatment, (2) evidence that the patient lacks capacity to consent to the study (as measured by a validated capacity to consent screener)[57]; or (3) evidence of current suicidal risk or harm to others (affirmative responses to question 4 and/or 5 on Paykel suicide questionnaire)[58].

D. STUDY SETTING

Participants for the randomized controlled trial were identified through recruitment in primary care clinics, community sites, and emergency departments, as well as from referrals from patients to

others in their social network who might benefit from the program. Recruitment was conducted between September 2014 and May 2016 in Boston, Madrid and Barcelona. Approval was obtained from the institutional review boards of participating institutions. We held a series of presentations with Directors and staff in the clinics and community sites, to introduce our staff and the study. We worked with the sites to determine optimal procedures for outreach and recruitment of patients.

E. SAMPLING, RECRUITMENT AND CONSENT

Research Assistants (RAs) approached potential participants in person, in the waiting room of clinics and community agencies. In certain sites, RAs took contact information for patients and followed up by phone to either screen or administer informed consent, depending on the permission granted by each institutional review board. In some cases, RAs called a list of patients, made available by clinical referral, directors of the site, or by access to electronic data.

E.1 Subject enrollment

Research staff first obtained informed consent from participants and then utilized the study screening to identify patients who were eligible for the study using the AC-OK short screener. We asked participants' permission to contact them through their PCP and collect information of two close contacts that had had the most frequent or stable contact with the participant over the past six months. We collected an address to send intervention materials and follow up letters and establish contact with the participant.

Patients who were NOT eligible received the short screener and the CAT-MH interview only, and were compensated $20. Patients who were eligible received the short screener and scheduled for a follow up baseline interview. The interview included instruments designed to identify mental health disorders, substance use problems, and HIV risk behaviors, as well as socio-demographics, cultural, contextual and social factors, medication use, chronic conditions, disability in daily activities, health literacy, language proficiency, access to health services, migration history, assessment of illness management/recovery and mindfulness. At baseline, they were also administered the CAT-MH interview and a urine test for drug metabolites to determine drug use. They were administered a capacity to consent form to ensure they understood and

could take part in the full trial. They were compensated $40-50 for the time spent in answering the assessment, depending on what assessment they were completing (see below).

Upon completion of baseline, participants were randomized into either the intervention or control condition, and began the trial. Additional interviews were administered by research assistants blind to study group at 2 months following baseline, 4 months, 6 months, and 12 months. At each interview a urine test was administered for drug metabolites.

Emergency protocol: We used an emergency protocol throughout the screening phase and in the trial. If participants responded affirmatively to questions in the screener or interview related to suicidal thoughts, they were administered the Paykel suicidality screener[5] after the interview. The emergency procedure was prompted if a patient endorsed a 4 or 5 on the Paykel suicidality screener over the past 30-days. The questions include: "Has there been a time in the last 30 days when you reached the point where you seriously considered taking your own life, or perhaps made plans how you should go about doing it?" = yes or "Has there been a time in the past 30 days/since the last interview when you made an attempt on your own life?" = yes. In Massachusetts, study staff connected the patient by phone or in person to the BEST emergency services team, which performs an assessment of patient safety and provides referrals in the event immediate care is needed. In the two sites in Spain, participants where referred to clinical staff overseeing the study, who assessed and helped connect with emergency care if needed. Participants with active suicidality could be contacted after 30 days for reassessment to see if they could safely participate in the trial if they were not actively receiving psychotherapy.

Certificate of Confidentiality: We received a Certificate of Confidentiality from NIDA for the US-based participant in this project.

F. RESEARCH PROCEDURES

F.1 Research Assistants

We recruited bilingual and bicultural study staff who would be engaging, non-judgmental, persistent, and not easily discouraged. Given the sensitivity around substance use and mental health issues and potential concerns about immigration status and discrimination, we selected

bilingual Spanish/English staff, who were also Latino and were sometimes born outside of the US or Spain, to build trust and familiarity with participants. In Barcelona, most of the study staff also spoke Catalan. All RAs received a comprehensive training to ensure they could conduct the interview effectively and made the participant feel comfortable. RAs followed 4 steps as part of their training 1) review of assessment questionnaire and materials, 2) remote training via WebEx, 3) conduct of at least two successful role play interviews of the sessions; and 4) Quality control of audio recording of first 2 patient interviews, including receiving detailed feedback from a study supervisor. During the study, all interviews were recorded and periodically evaluated under quality control procedures. A minimum of 15% (207 approximately) of all the interviews in the study were randomly selected, distributed to the quality control team and evaluated by the project coordinator of each site for feedback to the interviewer, and corrections if necessary.

F.2. Study Outcomes Measures

All participants in either condition took part in five research interviews (including baseline). Assessments were accompanied by administration of a urine sample for biological confirmation of substance use. Interview data was audio-recorded using a digital audio recorder and responses were inputted into tablet computers. To collect research data in the tablets, we work with Dimagi, a health care technology company, to implement data collection of the screeners and interviews via CommCare technology. This technology was installed on tablets, and made available to research assistants serving as interviewers so all information was kept secured on Dimagi servers.

Eligible patients were randomized into the intervention and control condition after baseline. They received follow up interviews and a urine screen at 2 months from baseline, 4 months from baseline, 6 months from baseline, and 12 months from baseline. They were compensated $30 (25**€** in Spain) per follow up interview, or $20 if they completed only half (primarily the outcome measures). For follow up at 6 and twelve months we compensated $50 (30**€** in Spain). We incorporated administration of the baseline and urine test to a random sample of patients who were NOT eligible, specifically to patients whose study ID ended with "7" but were not eligible. This helped in the analysis phase to identify differences between patients who were part of the intervention and who did not screen in. They were administered the short screener, then the

baseline interview, the CAT-MH as well as the urine test. In the event the interview took place by phone, we arranged for the participant to meet the interviewer at one of the study sites to administer the urine analysis before payment.

We collected the following measures as part of the study, including the outcomes.

Table 1. List of Assessment Measures/Variables

**Socio-Demographic variables**	
Demographics	Site (Boston/Madrid/Barcelona), age (18-34, 35-49, 50+), gender (male/female), race/ethnicity (White, Black, Indigenous/Native American, Hispanic/Latino/Caribbean, Mixed), education level (less than high school/HS Diploma, GED, vocational school, or more), Total Personal Income Last Year (< 15000 US Dollars or ≥ 15000 US Dollars)
**Clinical Measures (Outcome Measures bolded)**	
**Depression - Patient Health Questionnaire (PHQ-9)**	9-item screening questionnaire to determine severity of depressive symptoms. Internal consistency was α= 0.85. Spanish version has good agreement with independent mental health professional diagnostic (k = 0.74; overall accuracy, 88%; sensitivity, 87%; specificity, 88%) [59].
**Generalized Anxiety - General Anxiety Disorder 7-item screener (GAD-7)**	7-item screening tool and severity measure for generalized anxiety disorder. In Spanish, a cut-off point of 10 showed adequate values for sensitivity (86.8%) and specificity (93.4%); AUC statistically significant [AUC = 0.957-0.985; p < 0.001]; Internal consistency was α= 0.86 [60]
**Post-traumatic Stress Disorder - PTSD Checklist for DSM-5 (PCL-5)**	The PCL-5 is a 20-item questionnaire, corresponding to the *DSM-5* symptom criteria for PTSD. We consider 33 as a cutoff given the latest data. The internal consistency was α= 0.94 [61]
**Drug Abuse - Drug Abuse Screening Test (DAST-10)**	10-item self-report instrument that has been condensed from the 28-item DAST. It is designed for clinical screening of substance use. The DAST-10 yields a quantitative index of the degree of consequences related to drug abuse. (α= 0.87) [62]
**Alcohol Abuse – Alcohol Use Disorders Identification Test – C (AUDIT-C)**	World Health Organization screener for excessive drinking. 3-question screen that evaluates lifetime and past 30 day alcohol use behaviors. [63] (α=0.78)
**Benzodiazepines - Benzodiazepine Dependence Questionnaire (BDEPQ)**	The BDEPQ is designed to measure dependence on benzodiazepine tranquilizers, sedatives, and hypnotics. We selected 10 representative items from this questionnaire. (α = 0.89) [64]
**Alcohol – Addiction Severity Index Lite (ASI Lite) – Alcohol and Drug - Addiction Severity Index Lite (ASI Lite) - Drug**	Shortened version of the Addiction Severity Index (ASI) which obtains lifetime information about problem behaviors, as well as problems within the previous 30 days. We used drug and alcohol questions only (α=0.84 for alcohol and α=0.70 for drugs)[65]
**HSCL-20 – Hopkins Symptom Checklist-20**	The HSCL-20 is a symptom inventory for depression that indicates severity of psychological distress[66]. Internal consistency was α=0.94
**Any positive in Urine Test**	Using the DrugCheck for drug metabolites with a binary outcome (yes/no) for use of any of this five drug types: amphetamines, benzodiazepines, cocaine, methamphetamine, and marijuana

Smoking - Fagerstrom Test for Nicotine Dependence	The Fagerstrom Test is a standard instrument for assessing the intensity of physical addiction to nicotine, and designed to provide an ordinal measure of nicotine dependence related to cigarette smoking. It contains six items that evaluate the quantity of cigarette consumption, the compulsion to use, and dependence (α =0.75)[69].
Trauma Exposure - Brief Trauma Questionnaire (BTQ)	10-item self-report measure that examines experiences with potentially traumatic events that would meet Criterion A (serious injury/life threat/subjective response) for PTSD diagnosis per DSM-IV. It is derived from the Brief Trauma Interview[70]. Internal consistency was α =0.537.
**Cultural/Social Factors**	
Citizenship (Yes/No)	Immigration status was defined by whether the participant was a citizen or non-citizen.
Years in US/Spain	
Number of Home Visits in the Past 12 Months	
Discrimination Scale	Everyday Discrimination Scale[71] (α=0.82). The scale measures chronic and routine unfair treatment in everyday life. Adopted from the Detroit Area Study and National Latino and Asian-American Study (NLAAS). Participants were asked 9 questions pertaining to discrimination that affect Latinos and other minorities.
Ethnic Identity Scale	The 3-item Ethnic Identity Scale (α = 0.73), derived from the 35-item Cultural Identity Scale for Latino Adolescents[72], was used to gauge cultural identity. Questions ask about what culture or ethnic/racial groups an individual identifies with.
Intercultural/Family Conflict Scale	We used 3 items of the Family/Culture Stress subscale of the Hispanic Stress Inventory (HIS) (α=0.66), designed to measure family interference with personal goals, arguments with family members, and the breakdown of the family unit[73-75].
Sense of Belonging	One question derived from qualitative research from the research team in previous studies[76]. The question is: You feel like you don't belong either in your Latino country) or in the U.S.?
**Recruitment Site**	
Recruitment Site	Primary Care Clinics/Community Sites/Emergency Room/Patient Referrals

F.3. Quality control

The ILRP formed a quality control (QC) group to monitor the quality of the data collected over the course of this study. The group consisted of a quality supervisor (Ph. D), quality manager (M.S), quality coordinators (BFA, MSW) and interviewers. The role of the quality supervisor was to serve as a conduit between the quality manager and the Principal Investigator (PI) to convey questions or concerns. Quality coordinators oversaw reporting of any errors or questions the interviewers had while interviewing to the quality manager.

To ensure reliable data through quality control, interviews were assessed every three months during data collection from all three sites. To eliminate potential bias and capture a representative sample of the quality of the interviews, it was determined that every three months the quality coordinators would assess around 15% of all the interviews of the trial. This work entailed between 1-2 hours per interview and additional paperwork. We ran 9 batches of QC interviews. The first batch started on October 2014. We conducted QC on 578 out of 3564 screeners and interviews (16%). Moreover, additional to the 15% QC of all interviews, we worked on inter-site and cross-site reliability of feedback of cases. In addition to the fifteen percent of quality control data, we performed data cleaning activities on a regular basis to identify systematic errors, for example: duplicate identifiers, missing data and wrong skip patterns.

We created a qualitative standard report for the interviewers to report and correct the findings during QC. This document covers: gathering complete and correct information (participant ID, interviewer ID, date of interview and read all the questions, following the order and extra information), minimal noise and adequate pace (appropriate voice tone, adequate tone and without noise and minimal interruptions), ensure instruments work (identify tablet problems and Identify recording problems) and avoid mistakes (interviewer skips questions and interviewer finishes the interview "early").

F.4. HIV and STI testing (NIDA Supplement)

We conducted testing for HIV and sexually transmitted infections (Chlamydia and Gonorrhea) among enrollees of the clinical trial for both control and intervention condition. Testing was offered in person. In the intervention condition, clinicians and study staff offered testing at session 7, which focused on HIV/STI risk reduction. In the control arm, care managers and study staff offered participants testing at the second usual care call.

G. INTERVENTION

G.1. Treatment condition

The IIDEA intervention was designed for the treatment of depression, anxiety, PTSD, and substance use problems in Latino migrants over 18 years old. Substances of use include a range of drugs including stimulants (e.g., cocaine), opiates (oxycontin, heroin) and misuse of over the

counter and prescription drugs (including benzodiazepines), as well as alcohol. Comorbid mental health disorder and substance use has been found to be a risk factor for HIV, and thus the intervention also addresses prevention of risky behaviors that can lead to HIV/STIs. Patients with severe substance use disorders, which include symptoms of physical withdrawal and tolerance (physical dependence), were not considered appropriate for this treatment until they had received medically needed treatment (e.g., medical detoxification and stabilization) to avoid medical complications that can arise due to discontinuing drug or alcohol use during treatment.

Study staff first took part in a cultural adaption process to make the manual more accessible to diverse Latino populations in the US and Spain. The IIDEA intervention provides a structured cognitive behavioral therapy (CBT) program designed to treat symptoms of depression, anxiety and posttraumatic stress disorder (PTSD) with coexisting substance use problems. It incorporates elements of CBT for PTSD, depression, and anxiety that have been shown to be effective in adult populations. It also presents a range of mindfulness practices and tools inviting the participant to be present in what he/she is experiencing instead of moving reactively into avoidance. The cognitive behavioral strategies can help patients who are at risk of recurrent depression, anxiety or trauma and who could therefore benefit from practicing coping strategies to assist them in their current daily situations. The introductory sessions use motivational interviewing (MI) and motivational enhancement to prepare participants for treatment, but this is a general approach which can help throughout treatment. At each session, the clinician administers a series of questions to assess how the patient was doing in mental health and substance use symptoms. We also connected patients to an emergency responder if they displayed active suicidality based on these assessments.

To decrease barriers to engagement in the IIDEA intervention, we designed the program to be deliverable both in person and over the phone. To this end, we discussed important considerations for phone delivery during the training of study clinicians. For instance, we discussed the need to ask participants whether they were in a private place with no distractions, the importance of vocal intonation, referring to the participant manual, and verbal affirmations to convey empathy.

The manual integrates HIV prevention by addressing risky behaviors that can be related to substance use and mental health disorders. A NIDA supplement provided additional funding to best incorporate this component. Substance use is intricately linked to risk for HIV/STIs, not only through intravenous drug use but also by interfering with judgment, leading to risky sexual behaviors.

G.2. Study Clinicians, Clinician training, Fidelity and Supervision

Study Clinicians

The IIDEA intervention was administered by clinicians with at least a Master level of training (psychologists, social workers, counselors). We drew on the model of Clinician's role development to recruit, select and train clinicians who enjoy interacting with people, are computer literate, and have experience with behavioral health treatments. The clinicians were trained on research objectives, ethics in research design, patient privacy and confidentiality (standard HIPAA training), and on understanding the skills and objectives of the training manual.

We devoted substantial time to training the mental health clinicians to offer the clinical intervention at the study sites. We structured a process for weekly clinical supervision to ensure teams implemented the intervention in a similar manner across sites and held weekly calls across all site supervisors every week. At each site, a Care Manager helped track and facilitate patients' participation in the intervention.

Clinician training

Training consisted of at least 50 hours of didactic instruction and role plays in addition to ongoing evaluation through pilot cases and reinforcement of skills. The supervisors (licensed clinicians) aided in the training (and re-training if necessary) of the clinicians. Roles plays were used to demonstrate competency in administering the IIDEA intervention; clinicians completed two full practice rounds of sessions 1-10 (20 session's total -role plays) and at least two pilot cases that received the 10 sessions. If the local supervisor considered that the clinicians showed the requisite levels of proficiency in the overall intervention, the clinician could then begin the treatment in the clinical trial with eligible participants.

Fidelity

Several procedures were performed to ensure treatment fidelity and standardize delivery to minimize an interventionist effect, both within and across sites. We standardized the intervention by strict training and certification on the administration of the intervention (as above), and using the IIDEA training manual. Clinicians were required to fill out checklists of all intervention components delivered during a session and to document session length to monitor "dose" delivered. The supervisors performed a full fidelity check for the pilot cases and for first two trial cases. In addition, a random 15% sample of recordings and treatment fidelity checklists were reviewed by the supervisors who utilized structured evaluation forms. Clinicians were given weekly feedback in real time on fidelity and issues of clinical significance by supervisors who had listened to audio recordings of each of their cases. A random sample was selected to perform the fidelity check across supervisors in Boston, Madrid and Barcelona. The team listened to several cases and evaluated inter-rater agreement between supervisors using supervisor agreement.

Supervision

We established three types of supervision during the study to ensure adherence to the IIDEA manual and protocol: individual, group and supervision of supervisors.

In all sites, individual supervision consisted of weekly, one hour discussion of the performance of clinicians based on the supervisor's reviewed sessions. The feedback to clinicians focused on the review of the audio recording of completed sessions that the supervisor had listened before the meeting and used to rate the clinician. This intense coaching of the clinician continued until the clinician had completed two complete 10-session role plays, treatment of two pilot patients, and at least two intervention patients. Thereafter, the individual weekly supervision adopted a more global approach, and responded to specific concerns expressed by the clinician, with continued supervisor review of 15% audio recordings of sessions. This group supervision formed an essential part of the training/supervision process. Across all sites, the 90 minutes of supervision were used to: (a) provide clinicians with updates of the treatment, as per the weekly discussion of supervisors' sessions, (b) provide didactic training on any skill or intervention component that was considered necessary, including carrying out role-plays, (d) provide

collective feedback to clinicians from the supervisors' review of the previous week's cases; and (e) respond to any concerns and doubts the clinicians raised.

Weekly discussion of the supervisors was carried out as part of the weekly teleconference. During this time supervisors voiced concerns and questions about cases they were supervising, and the lead supervisors provided feedback and constructive criticism to facilitate deeper understanding of the components of the intervention. In addition, any adaptations to the treatment predicated on previous sessions were outlined.

G.3. EUC condition

Control patients continued their usual care routine with their primary care physician (PCP), if they had one. To address potential symptom attenuation, we contacted EUC participants 4 times to administer the same brief assessment used in the clinical sessions to see how the patient was doing in different areas. The control group was overseen by the care manager, who performed the EUC calls once every three weeks after the baseline interview. The care manager also assisted with the referral process to specialized substance use or mental health services, which was provided if the patient requested them.

H. ANALYTICAL METHODS

H.1. Analytical Methods – Data Preparation

Some variables had missing data, due to drop-outs, missing assessments, lack of response to questions, or participant assessment of a question as not applicable. To account for missing data, we used multiple imputation methods in Stata version 14.2 via the *mi impute chained* command[77]. The multiple imputations were carried out in three steps: first, we imputed the missing data for all the variable considered, creating 20 imputed datasets that each consist of 341 participants with four follow-up assessments per participant. Second, we ran analysis on each individually imputed data set; third, we aggregated the individual estimate to obtain final estimates and adjusted standard errors for the uncertainty due to imputation[78].Each imputation was carried out using the chained equations method[79]. In what follows, we refer to variables with missing data as incomplete variables and those with non-missing data as complete variables. Each incomplete variable x_j was specified as a conditional function (g_j) given the set of all other variables used in the imputation, comprising both incomplete ($x_1,\ldots,x_m$) and complete variables

(Z). We fitted the conditional model g_j to generate the predicted values of x_j using an iterative method. Specifically, each incomplete variable x_j was iteratively estimated and in each iteration, the variable x_j^t was then updated to x_j^{t+1} based on the conditional model. This updated variable was then used in the estimations of the other variables, following the conditional model specification: $x_j^{t+1} \sim g_j (x_j \mid x_j^{t+1},\ldots, x_{j-1}^{t+1}, x_{j+1}^{t},\ldots Z, \phi_j)$, for $j \in \{1..m\}$, where ϕ_j j were parameters of the conditional model g_j . These steps were repeated for all variables $x_1 ,\ldots, x_m$ and, after an initial burn-in phase, the procedure was stopped once convergence was reached. The variables used for imputation included outcome variables and their baseline measures, patient socio-demographics, clinical characteristics, and study design variables, such as site and intervention indicators, as well as dummy indicators for each follow-up assessment.

We used interval regressions to incorporate the theoretical bounds of the clinical outcome variables to increase the efficiency and accuracy of the imputation procedure, (e.g., we restricted the imputed values of PHQ-9 to lie within the 0 to 27 intervals). To impute the binary variables, (e.g. whether the urine drug test was positive), logistic regressions were used. Other conditional models were specified to be multiple linear regressions.

After running analysis on the twenty data sets we imputed via step 1, we combined these separate estimates from the different data sets to arrive at our final estimates.

Power Analyses: We calculated power in multivariate regression models, assuming a squared multiple correlation of 0.20 between the treatment indicator and 20 other covariates, a type I error of 0.05, and a two-sided comparison. Using intervention effect sizes and standard deviations from previous studies, we expected our projected sample (180 treatment and 180 control) to provide 80% and 98% power to detect statistically significant treatment group reductions in the HSCL-20 and PCL-5, respectively. Based on the above-mentioned pilot study finding that brief CBT decreased the percent of calendar days with any drug use and any marijuana use, we expected 90% power to detect treatment group improvements in cannabis abstinence, and 99% power to detect treatment group improvements in abstinence from any drug. No prior studies were identified to calculate power for detecting GAD-7 and ASI. We also assessed the power to test interaction effects between the intervention and individual-level

cultural factors. Assuming we have 25 variables in the model and the R-squared of the model is .20 with the interaction effects contributing .02 to the explanatory power of the model, then we will have 83% power to detect a statistically significant interaction coefficient in the full sample of 360 patients.

H.2. Data Analysis

First, we compared distributions of baseline characteristics between participants who received the IIDEA intervention and participants in the enhanced usual care group (EUC), to assess the balance of the observed covariates. For each follow-up assessment, we compared the baseline covariates to detect any statistically significant baseline differences between those who completed the assessment versus those who did not.

Second, to assess the effect of the intervention, we conducted an intent-to-treat (ITT) analysis, using a multilevel, multivariate regression model to assess changes in outcome variables over time in the treatment and control groups. To account for the nature of longitudinal data, our ITT analysis was carried out by fitting multilevel mixed-effects models to allow for valid variance calculation and statistical inference. The multilevel models we used included random effects at the patient level to account for within-patient correlations, and robust clustered standard errors to account for within-clinic correlations due to patients nesting within the same clinic. Letting Y_{it} denote the outcome measured at time t after the baseline for patient *I*, we estimated the following model:

$$(1)\quad Y_{it} = \beta_{0i} + \beta_1 Intervention_i + \beta_2 Time_t + \beta_3 Intervention_i * Time_t + \beta_4 (Time_t - t^*) + \beta_5 Intervention_i * (Time_t - t^*) + \beta_6 X_i + \varepsilon_{it}$$

where the patient-specific random intercept can be written as $\beta_{0i} = \alpha_{00} + \omega_{0i}$. We fitted linear models for continuous outcomes and logistic models for binary outcomes. Although scores on the ASI drug and ASI alcohol measures range from 0 to 1, participant scores on these measures were rescaled to a range of 0 to 100 prior to regression analyses (i.e., multiplied by 100). This adjustment was made to ensure meaningful regression estimates.

In the ITT analysis, individuals were assigned to the study arm to which they were randomized. Thus, $Intervention_i$ is equal to 1 if patient i is randomized to the intervention arm and 0

otherwise. $Time_t$ is a continuous measure of time capturing differences in months between assessment t and assessment $t - 1$. Because the intervention ended by the time of the 6-month assessment, we centered time variables at 6-month follow-up in the following way: $Time_t$ equals to -4 for 2-month follow-up, -2 for 4-month follow-up, 0 for 6-month follow up, and 6 for 12-month follow-up. To model the pattern of outcome changes over time, our primary analysis employed linear spline models to divide the time axis into two segments, and within each segment consider piecewise linear trends (e.g., having a different time trend before and after the intervention ended). Specially, we denote t* to be the month when the intervention stopped, i.e., 6 months after baseline and $(Time_t - t^*)$ to be the post-intervention time trend, which equals to $(Time_t - t^*)$ if $Time_t > t^*$ and 0 otherwise. This choice of linear spline models allows for the ability to test whether the time trends differ before and after the intervention was finished and that treatment effect could attenuate over time once participants did not receive more intervention. Since the time variable is centered at 6-month follow-up, the beta coefficient on $Intervention_i$ can be interpreted as the treatment effect on outcome levels evaluated at the end of the intervention. That is, testing for the significance of β_1 tests the hypothesis that the treatment was more effective than EUC in reducing substance use disorder and/or mental health problems, as evaluated at 6 months after the baseline. The coefficient of intervention by time interaction β_3 , tests whether the pattern of outcome over time would be no different between treatment and EUC groups. Similarly, β_5 tests whether changes in outcome responses over time were different between treatment and control groups after the intervention had ended. The constant term α_{00} in model (1) represents the average level of outcome variables in the control group measured at the end of the intervention. The term ω_{0i} denotes the patient-specific random effect and ε_{ij} denotes the residual error term. X_i includes the baseline response of outcomes to control for severe differences at baseline and site indicators to adjust for variations by location. Because a successful randomized control trial balances both known and unknown confounders between treatment and control groups, our primary ITT analyses did not control for other covariates. We tested the robustness of the results by further adjusting for current psychotropic medication use in a sensitivity analysis. The adjusted results did not differ from the main results. Our study sample was comprised of a broad clinical population, with a substantial number of patients who only had mild symptoms in substance use and mental health problems. To test the hypothesis that the intervention would be more effective among those with moderate to severe

symptoms, we extended model (1) to include an intervention by baseline severity interaction. Baseline severity variable was dummy-coded, with 1 if participant's baseline outcome score equals or exceeds a moderate level of symptoms and 0 otherwise. Thresholds of moderate severity level are: 0.1 for ASI-Alcohol and ASI-Drug in their original scales, being positive to at least 1 substance on the urine drug test, 10 for PHQ-9, 10 for GAD-7, 33 for PCL-5, 1.5 for HSCL-20, 35 for the composite mental score and 20 for the composite substance score. These severity thresholds are based on either published literature or on the 50th percentile of the baseline outcome scores (except for the ASI-Drug measure). Because the distribution of ASI-Drug in this sample is highly rightly-skewed, 75th percentile of the baseline outcome scores was chosen to be the severity threshold. An interaction between this baseline severity dummy and intervention was then added into Model (1). With this adjustment, β_1 now represents the intervention effect at 6-month follow-up for those who had mild symptoms. The coefficient of the baseline severity and intervention interaction measured the incremental effect of the treatment among participants with moderate to severe baseline symptoms.

In the context of multisite randomized control trial, we examined whether the intervention was equally effective at the three sites. To do this, we added an intervention by site interaction to Model (1) and tested if the interaction term was statistically significant, with Boston as the reference group. We also estimated whether intervention effects were the same among patients who received the most sessions by telephone vs. in-person. We recategorized participants into four mutually exclusive groups: 1) patients in the control arm; 2) intervention patients who received zero sessions, 3) intervention patients who received most sessions by telephone, and 4) intervention patients who received most sessions in-person. When there was a tie between number of sessions received by telephone vs. in-person, we randomly assigned the patient into either group. Finally, we replaced the dummy-coded intervention variable with this multi-group variable to refit the ITT model.

In secondary analyses, we used *dosage* (defined as number of treatment sessions received) as the independent variable of interest, categorized as dosage equal to zero (control group), 0-3 sessions (inadequate treatment for the intervention group), 4 or more sessions (adequate treatment for the intervention group). While this analysis no longer relies on random assignment, it serves to

provide confirmation of the results from the intent-to-treat analysis and provides further estimates of the magnitude of the intervention effects on the outcomes evaluated at 6 months after the baseline. Because the dosage analysis relies on actual treatment received, we used the original non-imputed sample for the analysis. To check the robustness of the *dosage* analysis, we conducted a third sensitivity analysis with an alternative categorization of treatment dosage. This alternative dosage variable has three categories: zero (control group), 0-5 sessions (intervention group) and 6 sessions or more (intervention group). We considered completion of treatment if the patient received 6 sessions or more, as 6 sessions cover the core components of the intervention (i.e. cognitive restructuring exercises, mindfulness practice, relapse prevention, etc.). Separating those with six or more sessions will allow us to examine the treatment effect for those who completed the core components of the intervention. We also performed separate sensitivity analyses to ensure the robustness of the results to alternative modeling strategies, estimation methods and how the missing data was handled.

Our first set of sensitivity analyses explored alternative methods for handling baseline response. Our primary analysis was carried out through analysis of covariance, which analyzes post-baseline responses, and makes an adjustment for the baseline response by including it as a covariate. In the sensitivity analysis, we retained the baseline response as part of the outcome vector and assumed the group means were equal at baseline, as is appropriate in a randomized control trial. The analytical data was extended to a longitudinal dataset, consisting of 5 repeated measurements per person, with baseline assessment included as an additional repeated assessment. Next, we re-estimated the model (1) with this new dataset. Since the baseline was added as additional time point, the $Time_t$ variable in this analysis, equals to -6 for baseline, -4, -2, 0 and 6 for the two, four, six and twelve-month assessments. We chose to present analysis of covariance as our primary results due to its potential efficiency gain. Because the baseline value has been obtained before any study intervention, i.e., the mean response that baseline is independent of treatment assignment, adjustment for baseline through covariance analysis will be more efficient, as it yields estimates of treatment effects with smaller standard errors[80].

Our primary ITT analysis modeled the nonlinear trend by fitting piecewise linear trends before and after treatment completion. We explored alternative modeling strategy in sensitivity analysis

where the post-treatment trend in the model (1) was replaced with a quadratic term of time trend to model the non-linear time trend. Linear spline models were finally chosen because they generally provide a flexible way to accommodate many non-linear trends that cannot be approximated by simple polynomials in time[80].

Next, we checked the robustness of the results with respect to estimation method. Our primary ITT analysis used multilevel mixed-effect models to account for inter-participant correlation due to repeated measurement. Our sensitivity analysis instead used generalized estimating equations (GEE). GEE estimation will produce unbiased estimates if the underlying correlation structure is correctly specified. However, it has the limitation of being less efficient than a properly specified mixed model and does not allow weights to vary within clusters/panels[80].

We also performed a sensitivity analysis using a propensity score weighting approach to balance the treatment and control groups on residual differences within each site after randomization. To do so, we estimated a logistic regression model of membership in the treatment group conditional on baseline measures and interactions between baseline measures and site, and generated the predicted probability (*phat*) of being assigned to the treatment group. In multivariate regression models, treatment group participants were given a weight of *1/phat* whereas control group participants were given a weight of *1*/1-*phat* to balance the groups on their predicted probability of being in the treatment group. Propensity score weights were then applied to all regression analysis. The weighted results remain similar to our main results.

Finally, we reran the *dosage* analysis with imputed data as a sensitivity check. In comparison to list-wise deletion, estimates of the imputed data from multiple imputation have slightly larger effect sizes (available from the authors upon request). This is because the intervention effects were greater among patients with moderate to severe symptomatology compared to those with mild symptomatology. Patients with missing observations, i.e., those who did not complete all follow-up assessments, had higher baseline drug symptom severity but where similar in baseline measures. Thus, by including these patients when using multiple imputation, the estimated intervention effects were amplified. Our primary analysis, which used non-imputed data and list-wise deletion, provides a conservative estimate of the actual effect of the treatment dosage.

I. SAFETY

I.1. Risks and Discomforts

We did not anticipate noteworthyrisks associated with the proposed study. Minimal risks included the possibility of discomfort when discussing mental health, substance use, or confidential HIV/STI-related problems with the clinicians (for intervention arm) or during research assessments (for both intervention and EUC groups). We indicated to participants in the treatment condition that they could become upset in discussing their frustrations dealing with behavioral health concerns or seeking adequate care for dealing with these problems. Participants being administered screening or research assessment instruments could experience mild emotional discomfort in responding to sensitive questions in the interview.

Another possible risk was that some participants may feel uncomfortable answering certain questions or may feel a burden of answering questions. Respondents were told during the research assessments that they had the option of terminating the interview at any time or not answering specific questions. The interviewers were instructed to implement short breaks during the interview if the respondent became fatigued or commented about the length of the interview.

Every precaution was taken to maintain all rights and privacy protections. A data release agreement was signed by all investigators who worked with the data in any way. Because the goal of the study was to report findings based on aggregate data, all individual information obtained was held strictly confidential. These inherent risks are typically assessed to be low in comparison to the long-term potential benefits from this type of research. If patients endorsed suicidality in either of the research assessments, research assistants followed the emergency protocol described above.

I.2. Benefits:

Participants in the intervention arm could possibly achieve decreased substance use, improved depressive, anxiety, and/or PTSD symptoms, decreased HIV/STI risk behaviors, and/or improvements in global functioning. Participants in the intervention arm, as compared to the EUC condition, might be better able to recognize and self-manage their behavioral problems,

as well as increase their ability to deal effectively with structural barriers (e.g., coping with cravings, stigma) that could impede them from entering and staying in behavioral treatment. We hoped that the results of the study could provide evidence of the effectiveness of the IIDEA intervention for Latinos migrants with co-occurring disorders and decrease the unmet need for services. Participants randomized into the EUC group also benefited from support by research staff such as coordination of primary care appointments and referral to behavioral health services and/or HIV/STI testing.

J. MONITORING, QUALITY ASSURANCE AND ETHICS

Collection protocols were established to ensure accuracy and quality of the data obtained from all participant interviews by research assistants and treatment sessions by clinicians. Research assistants have regular meetings and conference calls during which data quality is a primary issue. These calls provide a forum for RAs to discuss issues and concerns pertaining to data collection protocol as well as a time to provide feedback on completed sessions to clinicians. Training at the start of the program and ongoingly ensures RAs are prepared to skillfully conduct interviews and respond appropriately to patient needs. Quality of the clinicians' work with intervention participants has been explained above (see G.2. section).

J.1. Participant Confidentiality

We made make every effort to ensure that participant data was safely stored. Each participant in the study was assigned a unique ID number. All documents that included private information (i.e. names, addresses or telephone numbers) were separated from de-identified research materials. These items were stored by research staff in a locked file. Project staff were the only people with access to the locked filing cabinet and/or any information or files that linked the case number with any identifying information. No reports were made public using any names or identifying information. Computerized data was identified by ID number only. The de-identified information that was transferred between study sites was be stored on a secured central server.

As part of hospital and university network, all research sites protected research data per research regulations and site data-related regulations. Sites adhered to strict data safety guidelines, including implementing network firewalls, antivirus systems, internet screening, auditing systems and network intrusion detection systems. All research staff computers were password-protected and had standardized, full featured software and hardware configurations. Only authorized research staff had access to the data. Emails containing potentially sensitive patient information were encrypted. Research staff identified patients by ID number in emails unless it is necessary to include patient's protected information (i.e., when updating primary care clinicians in a crisis).

REFERENCES

1. Gulbinat W, Manderscheid R, Baingana F, et al. The International Consortium on Mental Health Policy and Services: objectives, design and project implementation. *International Review of Psychiatry.* 2004;16(1-2):5-17.

2. Kohn R, Saxena S, Levav I, Saraceno B. The treatment gap in mental health care. *Bulletin of the World Health Organization.* 2004;82(11):858-866.

3. Galea S, Ahern J, Tardiff K, et al. Racial/ethnic disparities in overdose mortality trends in New York City, 1990–1998. *Journal of Urban Health.* 2003;80(2):201-211.

4. Galvan FH, Caetano R. Alcohol Use and Related Problems among Ethnic Minorities in the United States. *Alcohol Research and Health.* 2003;27(1):87-94.

5. Office of National Drug Control Policy. *The Economic Costs of Drug Abuse in the United States,1992-2002.* Washington, DC:2004.

6. Compton WM, Thomas YF, Stinson FS, Grant BF. Prevalence, Correlates, Disability, and Comorbidity of DSM-IV Drug Abuse and Dependence in the United States: Results From the National Epidemiologic Survey on Alcohol and Related Conditions. *Arch Gen Psychiatry.* May 1, 2007 2007;64(5):566-576.

7. Hasin DS, Stinson FS, Ogburn E, Grant BF. Prevalence, correlates, disability, and comorbidity of DSM-IV alcohol abuse and dependence in the United States: results from the National Epidemiologic Survey on Alcohol and Related Conditions. *Archives of General Psychiatry.* 2007;64(7):830.

8. Alegria M, Vera M, Freeman Jr D, Robles R, Santos MC, Rivera CL. HIV infection, risk behaviors, and depressive symptoms among Puerto Rican sex workers. *American Journal of Public Health.* 1994;84(12):2000.

9. Bing EG, Burnam MA, Longshore D, et al. Psychiatric disorders and drug use among human immunodeficiency virus-infected adults in the United States. *Archives of General Psychiatry.* 2001;58(8):721.

10. Stein MD, Solomon DA, Herman DS, Anderson BJ, Miller I. Depression severity and drug injection HIV risk behaviors. *American Journal of Psychiatry.* 2003;160(9):1659-1662.

11. Carey MP, Carey KB, Maisto SA, Schroder KEE, Vanable PA, Gordon CM. HIV risk behavior among psychiatric outpatients: association with psychiatric disorder, substance use disorder, and gender. *The Journal of nervous and mental disease.* 2004;192(4):289.

12. Meade CS, Sikkema KJ. HIV risk behavior among adults with severe mental illness: A systematic review. *Clinical Psychology Review.* 2005;25(4):433-457.

13. Rounsaville BJ, Weissman MM, Crits-Christoph K, Wilber C, Kleber H. Diagnosis and Symptoms of Depression in Opiate Addicts: Course and Relationship to Treatment Outcome. *Arch Gen Psychiatry.* February 1, 1982 1982;39(2):151-156.

14. Daughters SB, Braun AR, Sargeant MN, et al. Effectiveness of a brief behavioral treatment for inner-city illicit drug users with elevated depressive symptoms: the life enhancement treatment for substance use (LETS Act!). *Journal of Clinical Psychiatry.* 2008;69(1):122-129.

15. MacInnes N, Handley S, Harding G. Former chronic methylenedioxymethamphetamine (MDMA or ecstasy) users report mild depressive symptoms. *journal of Psychopharmacology.* 2001;15(3):181.

16. Swendsen JD, Merikangas KR. The comorbidity of depression and substance use disorders. *Clinical Psychology Review.* 2000;20(2):173-189.

17. Grant BF, Stinson FS, Dawson DA, et al. Prevalence and co-occurrence of substance use disorders and independent mood and anxiety disorders: results from the National Epidemiologic Survey on Alcohol and Related Conditions. *Archives of General Psychiatry.* 2004;61(8):807.

18. Stewart SH, Karp J, Pihl RO, Peterson RA. Anxiety sensitivity and self-reported reasons for drug use. *Journal of Substance Abuse.* 1997;9:223-240.

19. Brook DW, Brook JS, Zhang C, Cohen P, Whiteman M. Drug use and the risk of major depressive disorder, alcohol dependence, and substance use disorders. *Archives of General Psychiatry.* 2002;59(11):1039.

20. Dixon L. Dual diagnosis of substance abuse in schizophrenia: prevalence and impact on outcomes. *Schizophrenia Research.* 1999;35:S93-S100.

21. Mueser KT, Bennett M, Kushner MG. Epidemiology of substance use disorders among persons with chronic mental illnesses. 1995.

22. Drake RE, Mueser KT, Clark RE, Wallach MA. The course, treatment and outcome of substance disorder in persons with severe mental illness. *American Journal of Orthopsychiatry.* 1996;66(1):42-51.

23. Kushner MG, Abrams K, Borchardt C. The relationship between anxiety disorders and alcohol use disorders: a review of major perspectives and findings. *Clinical Psychology Review.* 2000;20(2):149-171.

24. Burns L, Teesson M. Alcohol use disorders comorbid with anxiety, depression and drug use disorders:: Findings from the Australian National Survey of Mental Health and Well Being. *Drug and Alcohol Dependence.* 2002;68(3):299-307.

25. Buckner JD, Ecker AH, Proctor SL. Social anxiety and alcohol problems: The roles of perceived descriptive and injunctive peer norms. *Journal of anxiety disorders.* 2011.

26. Lasser K, Boyd J, Woolhandler S, Himmelstein DU, McCormick D, Bor DH. Smoking and mental illness. *JAMA: the journal of the American Medical Association.* 2000;284(20):2606.

27. Solway ES. The Lived Experiences of Tobacco Use, Dependence, and Cessation: Insights and Perspectives of People with Mental Illness. *Health and Social Work.* 2011;36(1):19-32.

28. Ismail K, Sloggett A, Stavola BD. Do common mental disorders increase cigarette smoking? Results from five waves of a population-based panel cohort study. *American journal of epidemiology.* 2000;152(7):651.

29. Tiet QQ, Mausbach B. Treatments for patients with dual diagnosis: a review. *Alcoholism: Clinical and Experimental Research.* 2007;31(4):513-536.

30. Wang PS, Aguilar-Gaxiola S, Alonso J, et al. Use of mental health services for anxiety, mood, and substance disorders in 17 countries in the WHO world mental health surveys. *The Lancet.* 2007;370(9590):841-850.

31. Cook B, Alegria M. Racial-Ethnic Disparities in Substance Abuse Treatment: The Role of Criminal History and Socioeconomic Status. *Psychiatric Services.* 2011;62(11):1273-1281.

32. Chisholm D, Flisher AJ, Lund C, et al. Scale up services for mental disorders: a call for action. *The Lancet.* 2007;370(9594):1241-1252.

33. Bijl RV, de Graaf R, Hiripi E, et al. The prevalence of treated and untreated mental disorders in five countries. *Health Affairs.* 2003;22(3):122-133.

34. Patel V, Thornicroft G. Packages of care for mental, neurological, and substance use disorders in low-and middle-income countries: PLoS Medicine Series. *PLoS medicine.* 2009;6(10):e1000160.
35. Alonso J, Angermeyer MC, Bernert S, et al. Use of mental health services in Europe: results from the European Study of the Epidemiology of Mental Disorders (ESEMeD) project. *Acta Psychiatrica Scandinavica.* 2004;109:47-54.
36. World Health Organization. *Global burden of mental disorders and the need for a comprehensive, coordinated response from health and social sectors at the country level.* Geneva, Switzerland: World Health Organization;2012.
37. Alegria M, Canino G, Rios R, Vera M, Rusch D, Ortega AN. Mental health care for Latinos: Inequalities in use of specialty mental health services among Latinos, African Americans, and non-Latino Whites. *Psychiatric Services.* 2002;53(12):1547-1555.
38. Llácer A, Amo JD, García-Fulgueiras A, et al. Discrimination and mental health in Ecuadorian immigrants in Spain. *Journal of epidemiology and community health.* 2009;63(9):766.
39. Mueser PR, Graves PE. Examining the role of economic opportunity and amenities in explaining population redistribution. *Journal of Urban Economics.* 1995;37:176-200.
40. Pellegrino A. Migration from Latin America to Europe: trends and policy challenges. *IOM Migration Research Series.* 2004.
41. Schiller NG, Basch L, Blanc CS. From immigrant to transmigrant: Theorizing transnational migration. *Anthropological quarterly.* 1995:48-63.
42. Instituto Nacional de Estadistica. *La población empadronada en España aumenta un 0,3% y alcanza los 47,1 millones de personas.* Madrid, Espana: Instituto Nacional de Estadistica;2011.
43. O'ferrall Gonzalez C, Crespo Linares M, Gavira Fernandez C, Crespo Benitez J. Inmigracion, ¿Estamos preparados los profesionales de la salud mental para este reto? *Interpsiquis.* 2003(1970):1-15.
44. Padilla B, Peixoto J. Latin American Immigration to Southern Europe. *Migration Information Source.* 2007. http://www.migrationinformation.org/Feature/display.cfm?ID=609. Accessed Feb. 16, 2012.
45. Acosta YD, de la Cruz GP. *The Foreign Born From Latin America and the Caribbean: 2010*: U.S. Department of Commerce: Economics and Statistics Administration;2011.
46. U.S. Department of Homeland Security. *2010 Yearbook of Immigration Statistics.* Washington, D.C.: Office of Immigration Statistics;2011.
47. Abraído-Lanza AF, Chao MT, Flórez KR. Do healthy behaviors decline with greater acculturation?: Implications for the Latino mortality paradox. *Social Science & Medicine.* 2005;61(6):1243-1255.
48. Alegria M, Mulvaney-Day N, Woo M, Torres M, Gao S, Oddo V. Correlates of past-year mental health service use among Latinos: results from the National Latino and Asian American Study. *American Journal of Public Health.* 2006:AJPH. 2006.087197 v087191.
49. Alegria M, Mulvaney-Day N, Torres M, Polo A, Cao Z, Canino G. Prevalence of psychiatric disorders across Latino subgroups in the United States. *American Journal of Public Health.* 2007;97(1):68.

50. Turner RJ, Lloyd DA, Taylor J. Stress burden, drug dependence and the nativity paradox among US Hispanics. *Drug and Alcohol Dependence.* 2006;83(1):79-89.
51. Pelissier B. Gender differences in substance use treatment entry and retention among prisoners with substance use histories. *American Journal of Public Health.* 2004;94(8):1418.
52. Horgan C, Skwara KC, Strickler G. *Substance Abuse: The Nation's Number One Health Problem: key indicators for policy*: Schneider Institute for Health Policy, Heller Graduate School, Brandeis University; 2001.
53. Collazos F. Inicis de tractament als centres de Barcelona segons país d'origen i droga consumida, 2010. Barcelona,Espana: Hospital Vall'Hebron; 2010.
54. Chang EC, Downey CA. *Handbook of Race and Development in Mental Health*: Springer Verlag; 2011.
55. World Health Organization. Lexicon of alcohol and drug terms published by the World Health Organization. http://www.who.int/substance_abuse/terminology/who_lexicon/en/. Accessed 2/10/12, 2012.
56. Cherry AL, Dillon ME. The AC-OK Cooccurring Screen: Reliability, Convergent Validity, Sensitivity, and Specificity. Journal of addiction. 2013;2013:573906.
57. Zayas LH, Cabassa LJ, Perez MC. Capacity-to-consent in psychiatric research: Development and preliminary testing of a screening tool. Res Soc Work Pract. 2005;15(6):545-56.
58. Paykel E, Myers J, Lindenthal J, Tanner J. Suicidal feelings in the general population: a prevalence study. Br J Psychiatry. 1974;124(582):460-9.
59. Kroenke K, Spitzer RL. The PHQ-9: a new depression diagnostic and severity measure. Psychiatr Ann. 2002;32(9):509-15.
60. Spitzer RL, Kroenke K, Williams JB, Löwe B. A brief measure for assessing generalized anxiety disorder: the GAD-7. Arch Intern Med. 2006;166(10):1092-7.
61. Blevins CA, Weathers FW, Davis MT, Witte TK, Domino JL. The posttraumatic stress disorder checklist for DSM□5 (PCL□5): Development and initial psychometric evaluation. J Trauma Stress. 2015;28(6):489-98.
62. Skinner HA. The drug abuse screening test. Addictive behaviors. 1982;7(4):363-71.
63. Bradley KA, DeBenedetti AF, Volk RJ, Williams EC, Frank D, Kivlahan DR. AUDIT-C as a brief screen for alcohol misuse in primary care. Alcoholism: Clinical and Experimental Research. 2007;31(7):1208-17.
64. Baillie A. Manual for the Benzodiazepine Dependence Questionnaire (BDEPQ): National Drug and Alcohol Research Centre, University of New South Wales; 1996.
65. McLellan A, Cacciola J, Zanis D. The Addiction Severity Index-“Lite”(ASI-“Lite”). Center for the Studies of Addiction, University of Pennsylvania/Philadelphia VA Medical Center. 1997.
66. Derogatis LR. The Hopkins Symptom Checklist (HSCL): a measure of primary symptom dimensions. 1974.
67. Brown KW, Ryan RM. The benefits of being present: mindfulness and its role in psychological well-being. Journal of personality and social psychology. 2003;84(4):822.
68. Carlson LE, Brown KW. Validation of the Mindful Attention Awareness Scale in a cancer population. Journal of psychosomatic research. 2005;58(1):29-33.

69. Heatherton TF, Kozlowski LT, Frecker RC, FAGERSTROM KO. The Fagerström test for nicotine dependence: a revision of the Fagerstrom Tolerance Questionnaire. Addiction. 1991;86(9):1119-27.
70. Schnurr P, Vielhauer M, Weathers F, Findler M. The brief trauma questionnaire. White River Junction, VT: National Center for PTSD. 1999.
71. Kim G, Sellbom M, Ford K-L. Race/ethnicity and measurement equivalence of the Everyday Discrimination Scale. Psychological assessment. 2014;26(3):892.
72. Felix-Ortiz M, Newcomb MD, Myers H. A multidimensional measure of cultural identity for Latino and Latina adolescents. Hispanic Journal of Behavioral Sciences. 1994;16(2):99-115.
73. Alegria M, Vila D, Woo M, Canino G, Takeuchi D, Vera M, et al. Cultural relevance and equivalence in the NLAAS instrument: integrating etic and emic in the development of cross-cultural measures for a psychiatric epidemiology and services study of Latinos. International journal of methods in psychiatric research. 2004;13(4):270-88.
74. Cervantes RC, Padilla AM, Salgado de Snyder N. The Hispanic Stress Inventory: A culturally relevant approach to psychosocial assessment. Psychological assessment: a journal of consulting and clinical psychology. 1991;3(3):438.
75. Cervantes RC, Padilla AM, De Snyder NS. Reliability and validity of the Hispanic Stress Inventory. Hispanic Journal of Behavioral Sciences. 1990;12(1):76-82.
76. Bird HR, Canino GJ, Davies M, Duarte CS, Febo V, Ramirez R, et al. A study of disruptive behavior disorders in Puerto Rican youth: I. Background, design, and survey methods. Journal of the American Academy of Child & Adolescent Psychiatry. 2006;45(9):1032-41.
77. StataCorp. Stata Statistical Software: Release 14. College Station, TX: StataCorp LP; 2015.
78. Rubin D. Multiple Imputation for Nonresponse in Surveys New York: John Wiley & Sons 1987.
79. StataCorp. Stata Statistical Software: Release 14. College Station, TX: StataCorp LP; 2015.
80. Fitzmaurice GM, Laird NM, Ware JH. Modeling the Mean: Analyzing Response Files. Applied longitudinal analysis. 998. Hoboken, NJ: John Wiley & Sons; 2012.
