# Supplementary material for: Evaluation of the Integrated Intervention for Dual Problems and Early Action Among Latino Immigrants With Co-occurring Mental Health and Substance Misuse Symptoms: A Randomized Clinical Trial
Source: JAMA Netw Open. 2019 Jan 11;2(1):e186927. doi: 10.1001/jamanetworkopen.2018.6927 (PMC6484537; doi:10.1001/jamanetworkopen.2018.6927)
Supplement: Supplement 3. — Data Sharing Statement [file jamanetwopen-2-e186927-s003.pdf]

Data Sharing Statement

Alegría. Evaluation of the Integrated Intervention for Dual Problems and Early Action Among Latino Immigrants With Co-occurring Mental Health and Substance Misuse Symptoms. *JAMA Netw Open*. Published January 11, 2019. 10.1001/jamanetworkopen.2018.6927

Data
Data available: No

Additional Information
Explanation for why data not available: This is a minority sample including participants with substance misuse. We are not able to release data as part of the publication, given the sensitivity of the data, and our agreement with the IRB that this information will not be shared.
